# Phylogenetic and Phylogenomic Analyses Reveal New Taxa in Clitocybaceae, with Notes on Poisonous Species and Spore Evolution

**DOI:** 10.3390/jof12080548

**Published:** 2026-07-23

**Authors:** You-Di Xu, Hai-Jiao Li, Chun-Ge Sheng, Zuo-Hong Chen, Zheng-Mi He

**Affiliations:** 1 Hunan Research Center of the Basic Discipline Developmental Biology, College of Life Sciences, Hunan Normal University, Changsha 410081, China; xuyoudi0112@163.com (Y.-D.X.); chenzuohong@263.net (Z.-H.C.); 2 National Institute of Occupational Health and Poison Control, Chinese Center for Disease Control and Prevention, Beijing 100050, China; lihaijiao715@126.com; 3 Mudanjiang Branch, Heilongjiang Academy of Agricultural Sciences, Mudanjiang 157000, China; shengchunge@sina.cn

**Keywords:** *Clitocybe*, *Collybia*, mushroom poisoning, phylogenomics, taxonomy

## Abstract

Clitocybaceae is a family of predominantly saprotrophic fungi with important edible and medicinal value. However, the edibility and toxicity of many species within this family remain unknown, posing a poisoning risk. In this study, 24 Chinese specimens, most of which were collected from poisoning sites, were identified. Phylogenetic analyses based on ITS and six loci (ITS, LSU, *TEF1*, *RPB1*, *RPB2*, and *ATP6*) and a phylogenomic analysis based on 485 single-copy genes were conducted to determine their placement within Clitocybaceae. Their morphological features were observed, particularly the spore surface. Based on the obtained results, a new genus with distinctly verrucose spores, *Allolepista*, is proposed to accommodate the clade that exhibits a very large genetic distance from its sister clade, *Lepista* s.str. A new species, *Clitocybe sinensis*, is described. Three new combinations, *A*. *crenata* (≡*L*. *crenata*), *A*. *benekei* (≡*Cli. benekei*), and *A*. *rhodotoides* (≡*L*. *rhodotoides*), are made. *Clitocybe violaceifolia*, originally described from the Pacific coast of the USA, is reported for the first time in China. *Collybia sinoborealis* and *Pseudolyophyllum liwanense* are found to be junior synonyms of *Col*. *violea* and *A*. *benekei*, respectively, while *Cli*. *lamoureae* and *Col*. *urbana* turn out to be synonymous with *Cli*. *truncicola*. Based on the symptoms observed in poisoning cases, *A*. *benekei* is found to cause gastroenteritis, while *Cli. sinensis*, *Cli*. *truncicola*, *Col*. *bisterigmata*, *Col*. *brunneoumbilicata*, and *Col*. *subtropica* lead to muscarinic syndrome. These results expand our understanding of the toxicity and species diversity of Clitocybaceae, providing a reference for public food safety.

## 1. Introduction

Clitocybaceae Vizzini et al. is a family classified within the suborder Tricholomatineae Aime et al. of the order Agaricales Underw [1]. The family was first referred to as “Clitocybées” by Roze [2], but this name had a French rather than a Latin ending and was thus invalid under Article 18.4 of the International Code of Nomenclature [3]. Van Overeem [4] invalidly introduced Clitocybaceae as a nomen nudum (Art. 38.1 of the ICN). The name was occasionally used informally or corresponded to the tribe Clitocybeae Fayod [5,6,7,8], prior to its valid publication by Vizzini et al. [9]. The name is now widely adopted and commonly used, with its monophyletic status strongly supported by recent phylogenomic and multi-locus phylogenetic analyses [10,11,12,13,14,15,16].

Clitocybaceae species are predominantly saprotrophic, with an extremely small minority being fungicolous (e.g., *Collybia tuberosa* (Bull.) P. Kumm. and *Dendrocollybia racemosa* (Pers.) R.H. Petersen & Redhead). They produce clitocyboid, lepistoid, or collybioid basidiomes with convex, umbonate, applanate, or depressed to funnel-shaped pilei, white, cream, pinkish, yellowish, or salmon spore deposit, and inamyloid basidiospores that are smooth, punctate, tuberculate, or verrucose to spinose [12,15]. Currently, the family comprises at least six representative genera, viz. *Clitocybe* (Fr.) Staude, *Collybia* (Fr.) Staude, *Dendrocollybia* R.H. Petersen & Redhead, *Lepista* (Fr.) W.G. Sm., *Pseudolyophyllum* Raithelh., and *Singerocybe* Harmaja, as supported by phylogenetic evidence accumulated over the past two decades [10,12,17,18,19,20,21]. Many species of this family are known to be edible (e.g., *Col*. *nuda* (Bull.) Z.M. He & Zhu L. Yang [22]) and/or medicinal (e.g., *Cli*. *nebularis* (Batsch) P. Kumm. [23]), while many others are known to produce the neurotoxin muscarine (e.g., *Cli*. *dealbata* (Sowerby) P. Kumm. [24]). These muscarine-containing species of Clitocybaceae have caused numerous mushroom poisoning incidents in Asia [25], Europe [26], and North America [27].

The type genus of Clitocybaceae is *Clitocybe*, for which the type species has been the subject of historical discussion. The designation of *Cli*. *nebularis* as the type species of *Clitocybe* was proposed by Earle [28]. As Earle followed the American Code [29], his typification selection is superseded unless previously confirmed (Art. 10.5 of the ICN). Donk [30] affirmed Earle’s selection, and this type is now widely accepted among contemporary scholars [6,31,32,33,34]. Bigelow [35] and Singer [36] objected to *Cli*. *nebularis*, proposing *Cli*. *clavipes* (Pers.) P. Kumm. and *Cli*. *gibba* (Pers.) P. Kumm. as type species, respectively. Both species are distantly related to most *Clitocybe* species and are now classified in the genera *Ampulloclitocybe* Redhead et al. and *Infundibulicybe* Harmaja, belonging to the families Omphalinaceae Vizzini et al. and Cuphophyllaceae (Z.M. He & Zhu L. Yang) Vizzini et al., respectively [15].

Extensive phylogenetic and phylogenomic analyses of Clitocybaceae by He et al. [12] revealed that *Clitocybe* fell into two unrelated clades. The first clade, named *Collybia* after its type species *Col*. *tuberosa*, includes the majority of traditionally recognized *Clitocybe* species, most of which contain muscarine. In contrast, the second clade, representing *Clitocybe* sensu stricto, does not produce muscarine and comprises only three *Clitocybe* species, including the type species of *Clitocybe*, *Cli*. *nebularis*. To prevent the nomenclatural instability that would arise from recombining hundreds of described *Clitocybe* species, He and Yang [11] proposed to conserve *Cli*. *phyllophila* (Pers.) P. Kumm. as the type for the genus. This lectotype lies within the core group of muscarine-containing clitocybes in the first clade, and it also belongs to the “tribe *Clitocybe*” as circumscribed in the protologue by Fries [37].

In the past three years, several taxonomic contributions have advanced the knowledge of Clitocybaceae. Martínez et al. [38] described a new species within the *Collybia* clade, *Cli*. *cedrelae* J.M. Suárez et al., from Argentina. Javeed et al. [39] reported an early diverging lineage within this family, *L*. *crenata*, from Pakistan. In China, Qi et al. [40] documented three newly discovered species of *Collybia*, and Zhang et al. [41] published seven new species belonging to *Collybia*, *Pseudolyophyllum*, and *Singerocybe*. Our recent field investigations in the wild and at mushroom poisoning sites across China have also revealed multiple taxa within Clitocybaceae. These taxa include a new species, a known species belonging to a novel genus, and six recognized species within the *Collybia* clade, one of which is newly recorded for China. In this study, we present morphological, phylogenetic, and phylogenomic evidence supporting these taxa, along with an assessment of their toxicity.

## 2. Materials and Methods

### 2.1. Specimens

A total of 24 specimens of Clitocybaceae were collected from Central (8), Northeastern (1), Northwestern (8), and Southwestern (7) China between 2018 and 2025, with 14 from mushroom poisoning sites in Guizhou [25,42], Hunan [43], Ningxia [42], and Yunnan [25]. Fresh basidiomes were dehydrated using heat at 45 °C. These specimens are deposited in the Mycological Herbarium of Hunan Normal University (MHHNU, Changsha, China) and the Herbarium of Kunming Institute of Botany, Chinese Academy of Sciences (HKAS, Kunming, China). Information regarding the specimens, including species name, GenBank accession number, voucher, and location, is provided in Table 1. Generic names used in this study are abbreviated as follows: “*A*.” for *Allolepista*, “*Atr*.” for *Atractosporocybe*, “*Cli*.” for *Clitocybe*, “*Col*.” for *Collybia*, “*D*.” for *Dendrocollybia*, “*L*.” for *Lepista*, “*P*.” for *Pseudolyophyllum*, and “*S*.” for *Singerocybe*.

### 2.2. Morphological Study

Macroscopic characters were described based on fresh specimens and photographs. The size of basidiomes, as determined by the width of the pileus, was described as small (<30 mm), medium-sized (30–50 mm), and large (>50 mm). The color codes cited in the descriptions are from Kornerup and Wanscher [44]. For the observation of microscopic structures, tissue sections of dried specimens were mounted in a 5% KOH solution or distilled water and observed under a light microscope. Basidiospores, basidia, and pileipellis were depicted through hand drawings.

In the description of basidiospores, the abbreviation [n/m/p] means that n basidiospores were measured from m basidiomes of p collections. The range notation (a)b–c(d) stands for the dimensions of basidiospores in which b–c contains a minimum of 90% of the measured values while a and d are the extreme values. The Q value represents the length/width ratio of basidiospores, and the Qm value is the average Q ± standard deviation. The shape of basidiospores was described following the terminology employed by Bas [45] and Vellinga et al. [46]. Melzer’s reagent and cotton blue were, respectively, employed with preheating to test the amyloidity and cyanophily of basidiospores. Scanning electron microscopy (SEM) was applied to observe spore ornamentation. Fragments of dry lamellae tissue samples were securely fastened to aluminum stubs and coated with gold palladium before being observed under a TESCAN CLARA Xplore 30 SEM (Tescan, Brno, Czech Republic).

### 2.3. DNA Extraction, PCR Amplification, Sequencing

Total genomic DNA was extracted using the Fungal DNA Mini Kit (Omega Bio-Tek, Norcross, GA, USA) following the manufacturer’s instructions. For the PCR amplifications, the following primers were employed: (1) ITS5 and ITS4 [47] were used for the internal transcribed spacer (ITS); (2) LR0R and LR5 [48] were used for the nuclear ribosomal large subunit (LSU); (3) EF1-983F, EF1-1953R and EF1-1567R [49] were used for the translation elongation factor 1-α (*TEF1*); (4) RPB1-Af and RPB1-Cr [50] or Collybia-RPB1-F and Collybia-RPB1-R [12] were used for the DNA-directed RNA polymerase II subunit 1 (*RPB1*); (5) RPB2-6F and RPB2-7.1R [51] or Collybia-RPB2-F and Collybia-RPB2-R [12] were used for RNA polymerase II subunit 2 (*RPB2*); (6) and ATP6-3 [52] and ATP6-6r [53] or Collybia-ATP6-F and Collybia-ATP6-R [12] were used for the ATP synthase subunit 6 (*ATP6*).

The PCR mixtures were composed of 1 × PCR buffer, 1.5 mM MgCl_2_, 0.2 mM dNTPs, each primer at 0.4 μM, 1.25 U of Taq polymerase (Yeasen, Shanghai, China), and 1 μL of DNA template in a total volume of 25 μL. Amplification reactions were performed with the following program: initial denaturation at 94 °C for 5 min, 35 cycles at 94 °C for 30 s, 50 °C (*ATP6*), 52 °C (LSU, *TEF1*, *RPB1*, and *RPB2*), or 54 °C (ITS and *RPB2*) for 30 s, 72 °C for 30 s (ITS, LSU, and *ATP6*) or 45 s (*TEF1*, *RPB1*, and *RPB2*), and a final extension at 72 °C for 10 min. All PCR products were subjected to electrophoresis on a 2% agarose gel, and the successful amplicons were sent to the Changsha branch of Youkang Biotechnology Co., Ltd. (Hangzhou, China) for sequencing.

### 2.4. Phylogenetic Analysis

**Figure 1:**
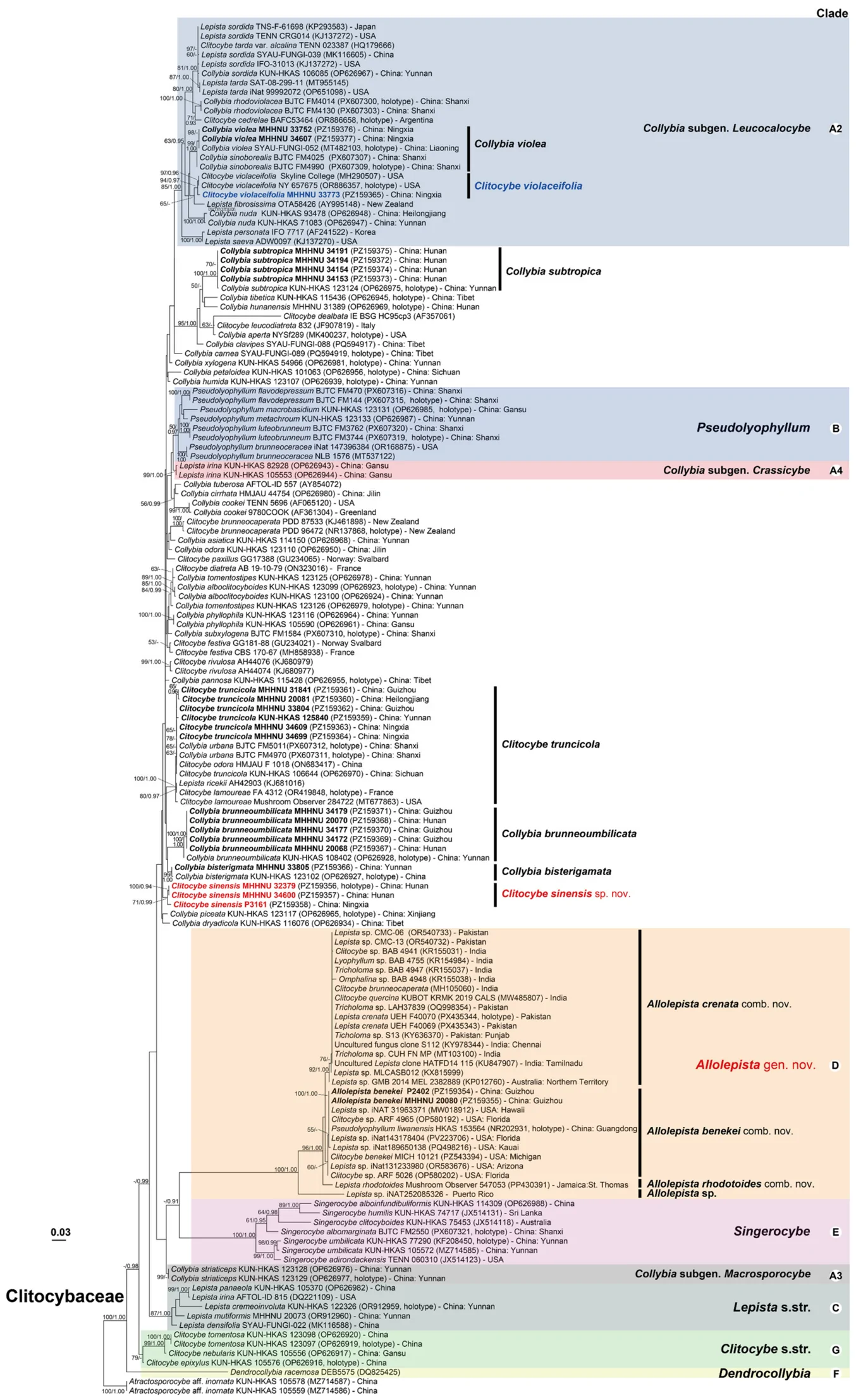
ML analysis of Clitocybaceae based on ITS sequence data, with *Atractosporocybe* aff. *inornata* as the outgroup. Bootstrap values (BPs) ≥ 50% from ML analysis and Bayesian posterior probabilities (PPs) ≥ 0.90 from BI analysis are shown at nodes. Samples studied in the present work are highlighted in bold, and new taxa are indicated in red. Accession numbers of sequences are given in parentheses, and their geographic origins are shown, if available.

**Figure 2:**
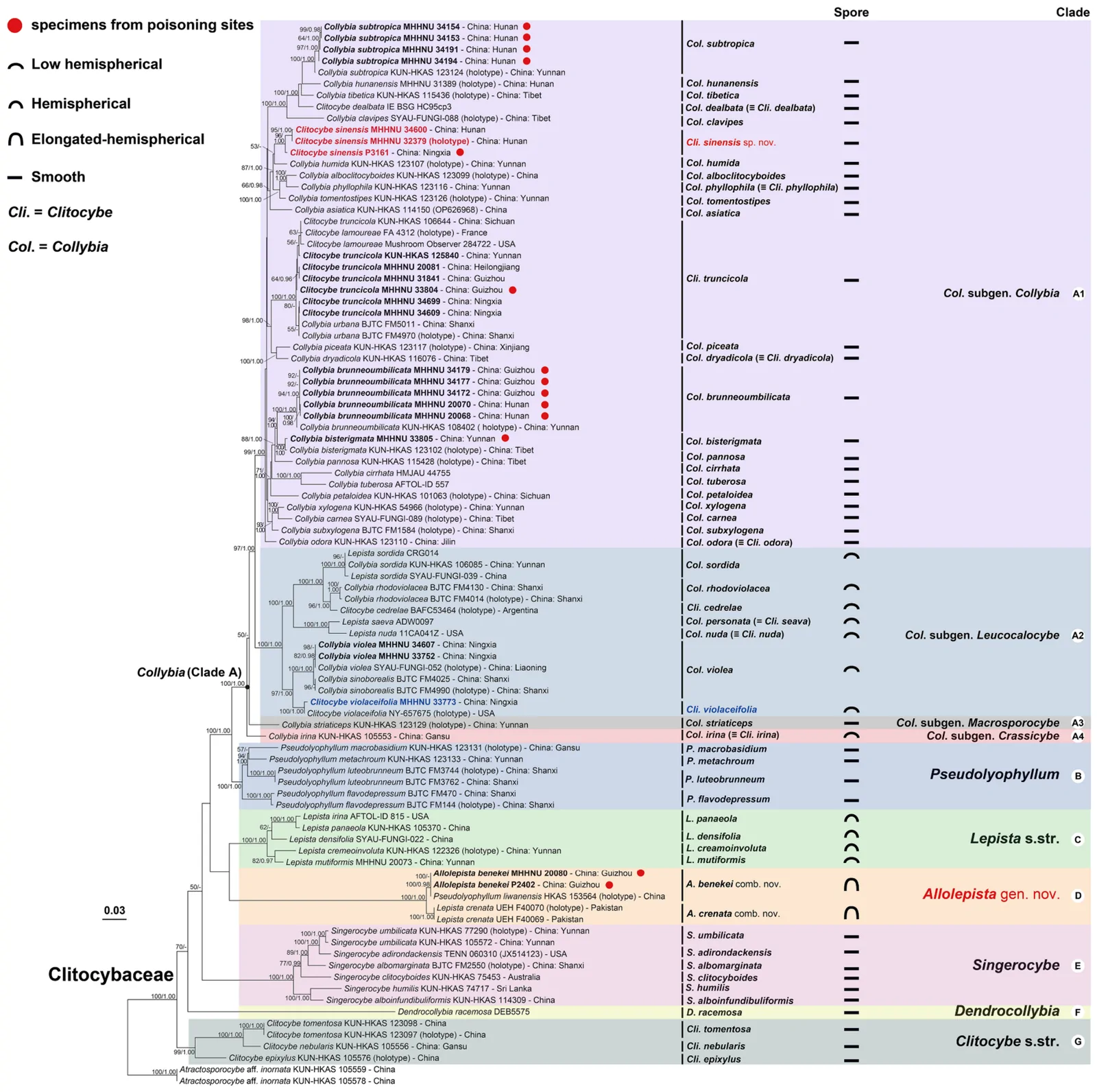
ML analysis of Clitocybaceae based on the combined ITS-LSU-*TEF1*-*RPB1*-*RPB2*-*ATP6* sequence data, with *Atractosporocybe* aff. *inornata* as the outgroup. Bootstrap values (BPs) ≥ 50% from ML analysis and Bayesian posterior probabilities (PPs) ≥ 0.90 from BI analysis are shown at nodes. Newly generated sequences, new taxa, and new Chinese records are highlighted in bold, red bold, and blue bold, respectively. The specimens from poisoning sites are marked with a red solid circle.

Two datasets were prepared for phylogenetic analysis. Dataset I comprised ITS sequences of representative species within Clitocybaceae (834 positions from 147 samples; Supplementary Material S1), while Dataset II contained sequences of ITS, LSU, *TEF1*, *RPB1*, *RPB2*, and *ATP6* (4538 positions from 93 samples; Supplementary Material S2). The accession and voucher numbers of these sequences are presented in Table 1 and Figure 1 and Figure 2. *Atractosporocybe* aff. *inornata* was selected as the outgroup because of its external position to Clitocybaceae [7,10].

Sequences were aligned using MAFFT v7.511 [54]. Sequence concatenation was performed using SequenceMatrix 1.7.8 [55]. In Dataset II, the intronic regions of *TEF1*, *RPB1*, and *RPB2* were manually excised (except for the conserved *RPB1*-intron 2), and the ambiguously aligned regions of ITS, LSU, and *RPB1* were removed using Gblocks v0.91b [56]. According to the AIC criterion in MrModeltest v2.4 [57], the GTR + I + G model was selected as the best-fit model for all partitions of Datasets I and II. Bayesian inference (BI) analysis was performed in MrBayes v3.2.7 [58] with the selected model, two simultaneous runs, four Markov Chain Monte Carlo (MCMC) chains, sampling every 100 generations, and a total of three or five million generations to ensure a standard deviation of split frequencies below the 0.01 threshold. The first 25% of generations were discarded as burn-in, and the convergence of runs was visually assessed using the trace function in Tracer v1.7.2 [59]. Maximum likelihood (ML) analysis with 1000 bootstrap replicates was computed in RAxML v8.0.20 [60], using the GTR + I + G model.

### 2.5. Genomic Library Preparation and NGS Sequencing

Genomic DNA was extracted using a Magnetic Plant Genomic DNA Kit (Tiangen Biotech, Beijing, China), following the manufacturer’s protocol. DNA was quantified using a Qubit 3.0 Fluorometer (Life Technologies, Carlsbad, CA, USA), and its integrity was assessed by electrophoresis on 1% agarose gel. A total of 0.2 μg DNA was fragmented by Covaris LE220R-plus (Covaris, Woburn, MA, USA) to a size of 350 bp. DNA fragments were end-polished, A-tailed, and ligated with the full-length adapter for Illumina sequencing, followed by PCR amplification [61,62]. DNA purity was assessed using the AMPure XP system (Beckman Coulter, Beverly, MA, USA). Library quality was assessed using the Agilent 5400 system (Agilent Technologies, Santa Clara, CA, USA) and quantified by real-time PCR. The library was sequenced on an Illumina Novaseq X plus platform (Illumina, San Diego, CA, USA) with a PE150 strategy in Novogene Bioinformatics Technology Co., Ltd. (Beijing, China). The sequencing data generated from our sample amounted to 6 Gbase.

### 2.6. Data Filtering and Genome Assembly

Original fluorescence image files were converted to raw data by base calling. FastQC v0.11.9 [63] was used to assess the quality of the raw data. Fastp v1.0 [64] was used for quality control and preprocessing of Fastq files. Genome assembly was performed with SPAdes v3.13.1 [65] using automatic k-mer selection based on read length. The completeness of the assembled genome was evaluated using BUSCO v5.2.2 [66].

### 2.7. Phylogenomic Analysis

**Figure 3:**
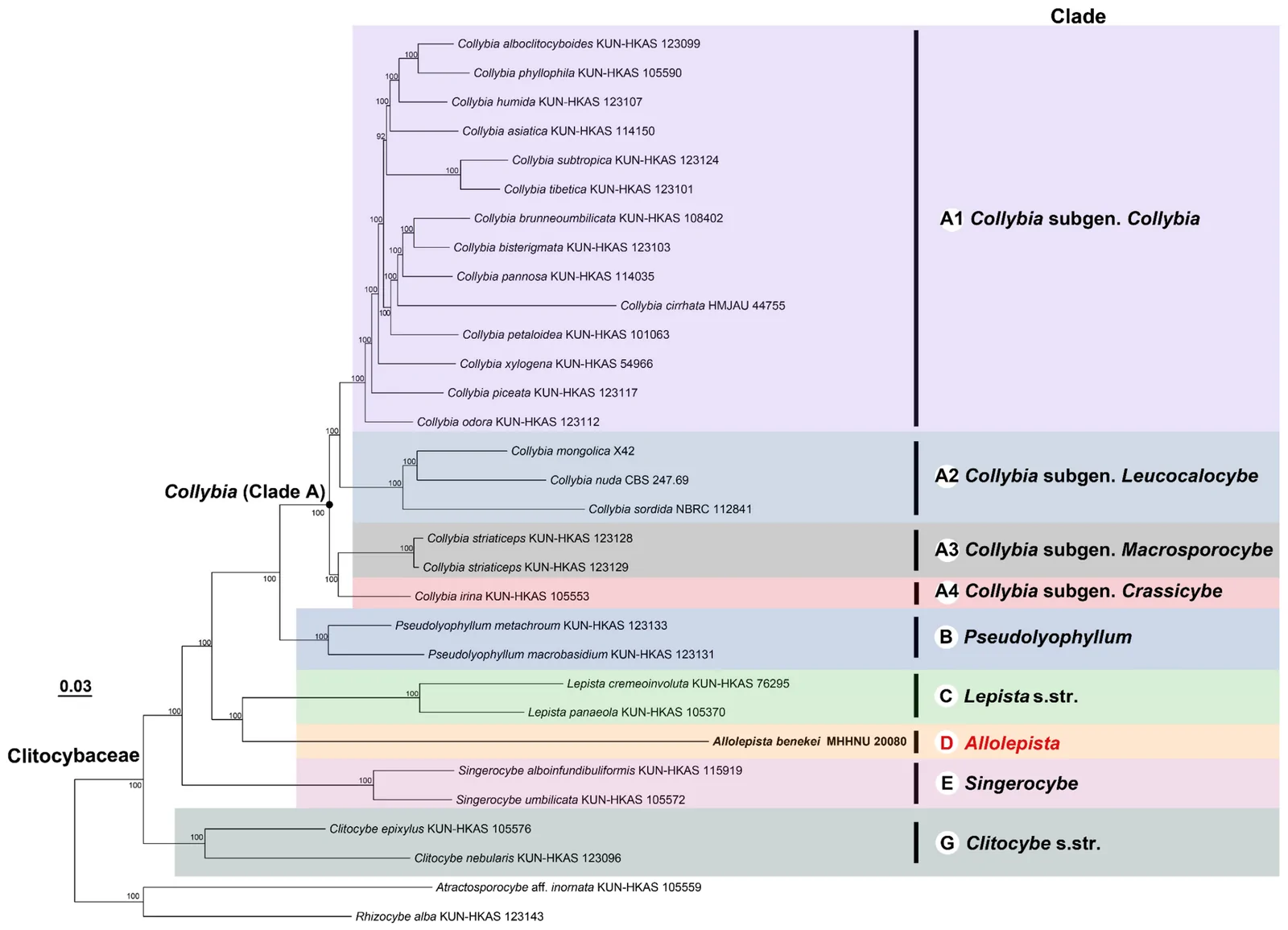
ML analysis of Clitocybaceae based on 485 single-copy genes showing the position of *Allolepista* (marked in red), with *Atractosporocybe* aff. *inornata* and *Rhizocybe alba* as outgroups.

Using BUSCO analysis, single-copy genes were retrieved by referring to the Agaricales_obd10 lineage dataset [https://busco-data.ezlab.org/v4/data/lineages/ (accessed on 7 December 2025)] [67]. The set of 485 single-copy genes employed by He et al. [12] was adopted. One sample of *A*. *benekei* (MHHNU 20080) in this study and 30 samples from He et al. [12] (Figure 3) were used for analysis. Amino acid sequences were aligned using MAFFT, and conserved sequences were extracted using Gblocks. The resulting sequences from these samples were concatenated to form Dataset III (486,149 amino acid sites; Supplementary Material S3). ML analysis with 1000 bootstrap replicates was performed with RAxML, using the JTT + G + I + F model [68].

## 3. Results

### 3.1. Phylogenetic and Phylogenomic Analysis

As shown in the ITS phylogenetic analysis of Clitocybaceae (Figure 1), the family is recognized as comprising the following genera: *Collybia* [type: *Col*. *tuberosa*], *Pseudolyophyllum* [type: *P*. *metachroa* (Fr.) Raithelh.] (Clade B: 50% BP, 0.97 PP), *Lepista* [type: *L*. *densifolia* (J. Favre) Singer & Clémençon] (Clade C: 87% BP, 1.00 PP), *Singerocybe* [type: *S*. *viscida* Harmaja (not sequenced)] (Clade E: 100% BP, 1.00 PP), *Dendrocollybia* [type: *D*. *racemosa*] (Clade F), and *Clitocybe* [type: *Cli*. *nebularis*] (Clade G: 79% BP, 0.89 PP). Clade D, with full support (100% BP, 1.00 PP), represents a different generic clade, which we name *Allolepista*. Nineteen of the analyzed specimens cluster together with the holotypes of *Col*. *violea*/*Col*. *sinoborealis* (99% BP, 1.00 PP), *Cli*. *violaceifolia* (94% BP, 0.97 PP), *Col*. *subtropica* (100% BP, 1.00 PP), *Col*. *brunneoumbilicata* (100% BP, 1.00 PP), *Col*. *urbana*/*Cli*. *lamoureae* (100% BP, 1.00 PP), and *Col*. *bisterigmata* (99% BP, 1.00 PP). Two specimens (MHHNU 20080 and P2402) cluster together with the holotype of *P. liwanense* (HKAS 153564) and seven American specimens, forming a lineage (55% BP, 0.55 PP) that is sister to the lineage containing *L. crenata* (92% BP, 1.00 PP). These two lineages are further united with *L*. *rhodotoides* within *Allolepista*. Three specimens (MHHNU 32379, 34600, and P3161) did not match any known sequenced lineages and form a new lineage named *Cli*. *sinensis* (71% BP, 0.99 PP).

The six-locus phylogenetic analysis (Figure 2) fully recovers *Collybia* (Clade A: 100% BP, 1.00 PP) and *Pseudolyophyllum* (100% BP, 1.00 PP) as separate clades and further resolves *Collybia* into four subclades: subgen. *Collybia* (A1: 99% BP, 1.00 PP), *Leucocalocybe* (X.D. Yu & Y.J. Yao) Z.M. He & Zhu L. Yang (A2: 100% BP, 1.00 PP), *Macrosporocybe* Z.M. He & Zhu L. Yang (A3), and *Crassicybe* Z.M. He & Zhu L. Yang (A4). The new species *Cli*. *sinensis* falls within subgen. *Collybia* and clusters with *Col*. *humida* Z.M. He & Zhu L. Yang, with weak support (53% BP, 0.73 PP). *Allolepista* forms a sister clade to *Lepista* s.str., but this relationship lacks statistical support.

To determine the exact phylogenetic placement of *Allolepista* within Clitocybeceae, a phylogenomic analysis based on 485 single-copy genes was performed. The resulting phylogram (Figure 3) confirms that *Allolepista* and *Lepista* s.str. are sister clades with 100% bootstrap support.

### 3.2. Taxonomy

**Figure 4:**
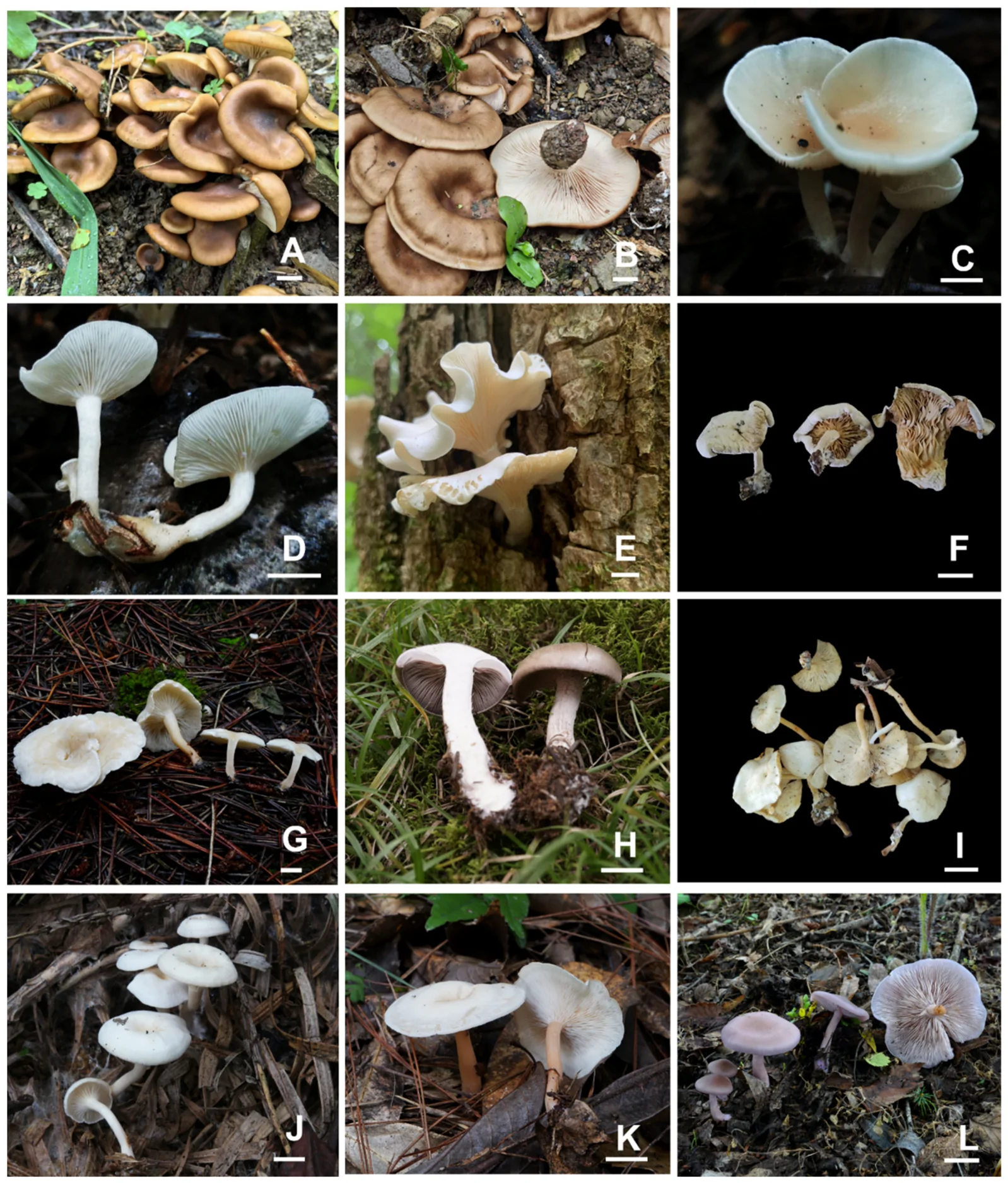
Basidiomes of the identified species. (**A**,**B**) *Allolepista benekei* (MHHNU 20080 and P2402, respectively); (**C**,**D**) *Clitocybe sinensis* MHHNU 32379; (**E**–**G**) *Cli*. *truncicola* (MHHNU 20081, MHHNU 33804, and MHHNU 34609, respectively); (**H**) *Cli*. *violaceifolia* (MHHNU 33773); (**I**) *Collybia bisterigmata* (MHHNU 33805); (**J**) *Col*. *brunneoumbilicata* (MHHNU 20070); (**K**) *Col*. *subtropica* (MHHNU 34153); (**L**) *Col*. *violea* (MHHNU 34607). Bars = 1 cm.

***Allolepista*** Z.M. He & Y.D. Xu, gen. nov. (Figure 4A,B).

**Fungal names.** FN 573727.

**Diagnosis.** Similar to *Lepista* s.str., but differs in its spores with more strongly developed verrucae that show no dimples under SEM.

**Etymology.** “*Allo*-“ (Gk.), different; “*lepista*” (Lat.), referring to the genus *Lepista*; that is, a genus similar to, yet distinct from, *Lepista*.

**Type species.** *Allolepista benekei* (H.E. Bigelow & A.H. Sm.) Z.M. He & Y.D. Xu.

**Description.** Habit clitocyboid to tricholomatoid. Basidiomes medium-sized to large. Veils absent. Pileus convex to applanate with depressed center, white, whitish, cream, brown, or other leather-like in color, not hygrophanous. Lamellae adnate to adnexed, crowded, cream to pinkish or brownish. Stipe central, cylindrical, thick. Basidiospores strongly verrucose, inamyloid, and cyanophilic. Basidia clavate, four-spored. Lamella trama regular to subregular. Cheilocystidia absent or present. Pleurocystidia absent. Pileipellis a xerocutis. Clamp connections present.

**Ecology.** Usually Gregarious, also caespitose, on humus or abandoned termite nest.

**Distribution.** Known from Asia, North America, and Oceania.

**Figure 5:**
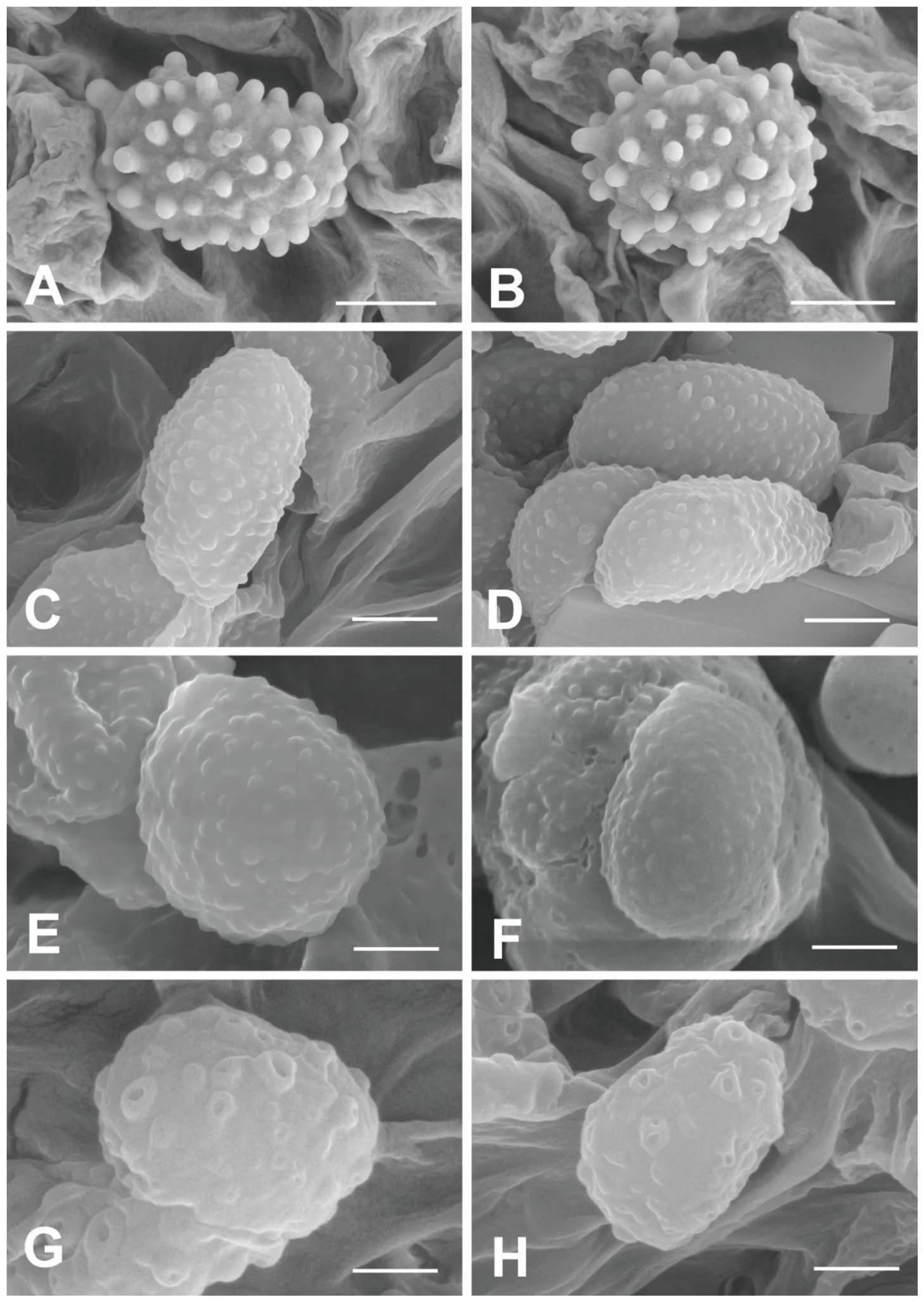
Basidiospores under SEM. (**A**,**B**) *Allolepista benekei* (MHHNU 20080, holotype); (**C**,**D**) *Clitocybe violaceifolia* (MHHNU 33773); (**E**,**F**) *Collybia violea* (MHHNU 33752); (**G**,**H**) *Lepista panaeola* (KUN-HKAS 105370). Bars: (**A**,**B**) = 3 μm, (**C**–**H**) = 2 μm.

**Notes.** *Allolepista* is proposed here to name a recently discovered lineage within Clitocybaceae reported by Javeed et al. [39], which corresponds to Clade D in our phylograms (Figure 1, Figure 2 and Figure 3). The phylogenomic analysis (Figure 3) fully confirmed that *Allolepista* is most closely related to *Lepista* s.str. The two genera both produce lepistoid basidiomes and verrucose spores. However, the spore verrucae of *Allolepista* are higher and do not show dimples compared to those of *Lepista* s.str. [21,69] (Figure 5G,H). The mature lamellae of *Allolepista* are adnexed to emarginate, while those of *Lepista* s.str. are adnate to decurrent [12].

**Figure 6:**
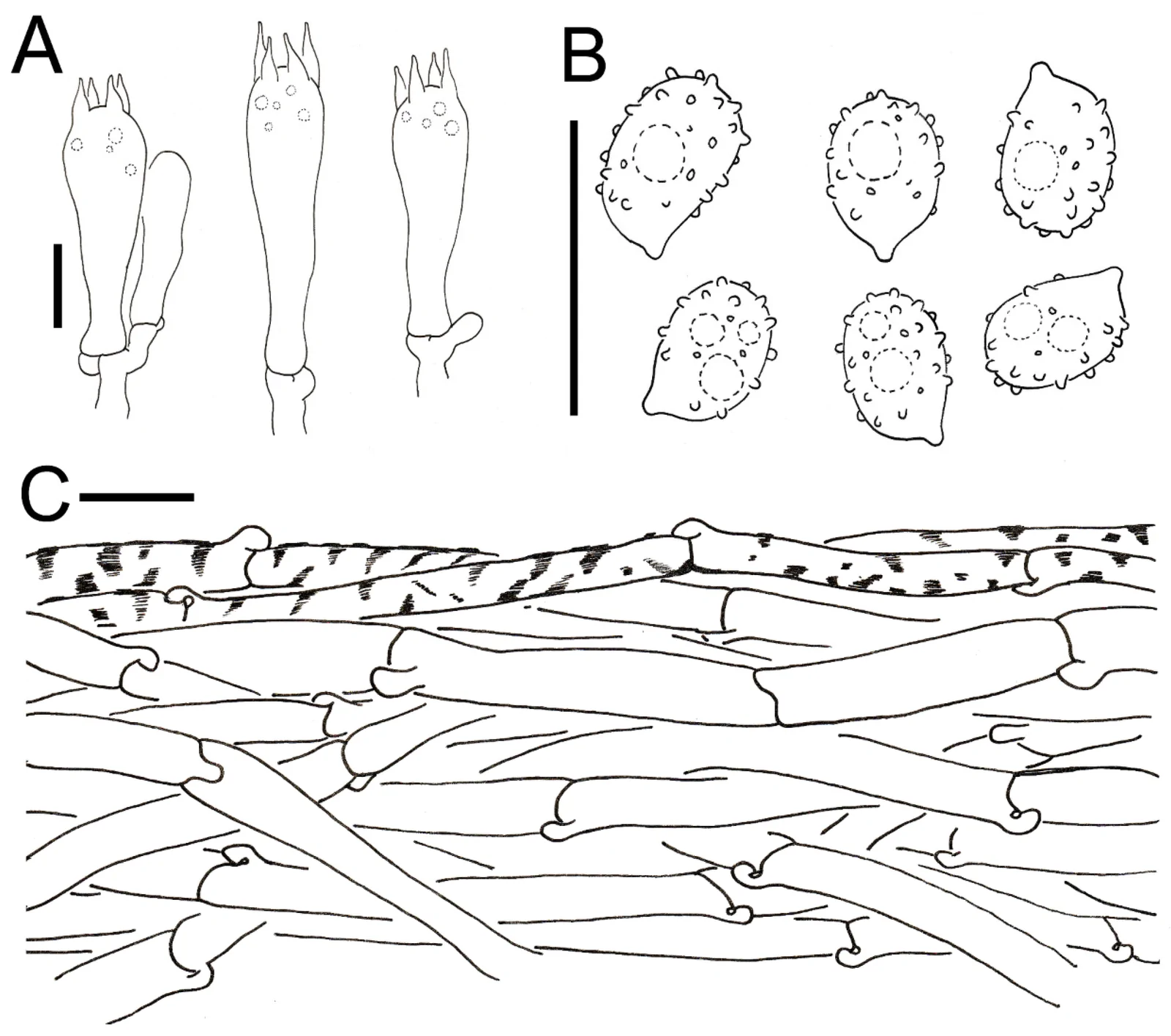
Microscopic features of *Allolepista benekei* (MHHNU 20080). (**A**) Basidia; (**B**) Basidiospores; (**C**) Pileipellis. Bars = 10 μm.

***Allolepista benekei*** (H.E. Bigelow & A.H. Sm.) Z.M. He & Y.D. Xu, comb. nov. (Figure 4A,B, Figure 5A,B and Figure 6).

**Fungal names.** FN 573729.

**Basionym.** *Clitocybe benekei* H.E. Bigelow & A.H. Sm., *Michigan Bot.* 9: 30 (1970).

= *Pseudolyophyllum liwanense* Kun L. Yang, Jia Y. Lin & Z.Chao Liu, in Yang, Lin & Li, *Phytotaxa* 746(1): 87 (2026).

**Description.** Basidiomes medium-sized to large, clitocyboid. Pileus 40–60 mm in diam, at first convex, then applanate, finally umbilicate to slightly infundibuliform; surface yellowish brown (5A3–5), brown (5C4–6) to grayish brown (5E5–7), felty at center, not hygrophanous; context white (1A1), 4–5 mm thick. Lamellae sinuate to emarginate, pale cream (2A2) to pinkish (5A2), yellowish brown (4A3) when dry, crowded, 3–6 mm high, in one or two tiers. Stipe 25–30 × 10–20 mm, central, cylindrical, solid, with longitudinally white (1A1) fibrils on a yellowish brown (4A3) background; base white (1A1) tomentose.

Basidiospores [60/2/2] 6–6.5(7) × 3–4(5) μm, Q = (1.40)1.50–1.88(1.59), Qm = 1.60 ± 0.11, ellipsoid to elongate, inamyloid, cyanophilic, pale brown, always single, with crowded, elongated-hemispherical verrucae measuring 0.4–0.8 × 0.3–0.7 μm (height × width; undimpled apically under SEM). Basidia 30–45 × 9–10 μm, clavate to broadly clavate, four-spored, thin-walled; sterigmata up to 6 μm long. Cystidia absent. Lamella trama regular to subregular, composed of thin-walled hyphae 3–15 μm wide. Pileipellis a cutis composed of subparallel to slightly interwoven hyphae 3–15 μm wide, with brown incrusting pigment. Stipitipellis a cutis composed of parallel, thin- to slightly thick-walled (about 0.5 μm), cylindrical hyphae 2–8 μm wide. Clamp connections present in all parts of the basidiome.

**Ecology.** Gregarious or caespitose, on humus, mainly in subtropical zones, also in temperate and tropical zones.

**Distribution.** Known from China (Guizhou and Guangdong Provinces) and the USA (Arizona, Florida, Hawaii, and Michigan).

**Specimens examined.** China, Guizhou Province, Renhuai County, Yanjin West Road, on humus-rich soil, at 27.79354° N, 106.36728° E, alt. 871 m, 27 June 2025, H.J. Li, MHHNU 20080; Renhuai County, Yanjin West Road, on humus, at 27.77693° N, 106.38154° E, alt. 760 m, 27 June 2025, H.J. Li, P2402.

**Notes.** Our ITS phylogeny (Figure 1) shows that our two Chinese sequences cluster together with the holotype sequence of “*P. liwanense*” (NR202931) from China and seven American sequences determined as “*Lepista*” (MW018912, PV223706, PQ498216, and OR583676), “*Clitocybe*” (OP580192 and OP580202), or *Cli. benekei* H.E. Bigelow & A.H. Sm. (PZ543394). These ten sequences are clearly conspecific, with pairwise identity values ranging from 99.34–100%. They were derived from mushroom samples collected in Guizhou, Guangdong, China, and Arizona, Florida, Hawaii, and Michigan, USA, which provides evidence for the widespread occurrence of this species across temperate to tropical zones in the Northern Hemisphere.

Our Chinese specimens exhibit a yellowish-brown pileus, cream to yellowish-brown lamellae, a whitish-fibrillose solid stipe, regular to subregular lamella trama, and distinctly verrucose brownish spores measuring 6–7 × 3–5 μm (5.5–7 × 4.5–5 μm in Bigelow and Smith [70]; 5.5–6 × 3.5–4 μm in Yang et al. [71]). These morphological features are fully consistent with the protologue of *Cli*. *benekei* published in 1970 [70]. Our photographs (Figure 4A,B) are also consistent with the historical images of this species [35,70,72]. The sequence identified as *Cli*. *benekei* was isolated from the specimen MICH 10121 collected near Mt. Clemens, Macomb County, Michigan, which is the same locality as the holotype MICH Smith 77176 [70]. Based on the above evidence, we identify the Chinese collections as the earlier name *Cli*. *benekei* and transfer it to *Allolepista* as *A*. *benekei* in light of its phylogenetic placement. The name *P*. *liwanense*, recently published by Yang et al. [71] in 2026, is not supported by our analysis because this species is phylogenetically distant from the smooth-spored *Pseudolyophyllum* (Figure 2). Instead, it is morphologically and molecularly identical to *A*. *benekei* and is, therefore, treated as a synonym.

*Allolepista benekei* is somewhat morphologically similar to brown-colored *L*. *subalpina* (Bigelow & Smith) Harmaja and *L*. *subisabellina* (Murrill) Kreisel. *Lepista subalpina* is a rare species found in the USA and Canada and differs from *A*. *benekei* by its occurrence in autumn, the absence of incrusting pigment in the pileipellis, and basidiospores that are often in tetrads and ornamented with small and distant verrucae [35]. *Lepista subisabellina*, only known from the Caribbean Region, differs from *A*. *benekei* by its pink spores with minute short prickles [73]. *Allolepista benekei* differs from *A*. *crenata* [39] by its fuscous pileus, larger basidiospores and basidia, and the absence of cheilocystidia.

The two specimens of *A*. *benekei*, MHHNU 20080 and P2402, were collected from the site of a mushroom poisoning in Guizhou Province, China, in 2025 [42]. The patients showed only symptoms of nausea, vomiting, diarrhea, and abdominal pain, suggesting that *A*. *benekei* is a gastroenteritis-causing species.

***Allolepista crenata*** (Javeed & Kiran) Z.M. He & Y.D. Xu, comb. nov.

**Fungal names.** FN 573728.

**Basionym.** *Lepista crenata* Javeed & M. Kiran, in Javeed, Kiran & Vizzini, *New Zealand J. Bot.* 64: 2 (2026).

**Notes.** In our analysis, *L*. *crenata* is phylogenetically part of the genus *Allolepista*, rather than *Lepista* s.str. (Figure 1 and Figure 2). Accordingly, it is combined in *Allolepista*. The ITS analysis (Figure 1) also shows that 15 GenBank sequences exhibit very low genetic divergence from those of *A*. *crenata* and should, therefore, be assigned to this species. The geographic distribution of these sequences shows that this species is not restricted to Pakistan [39] but also occurs in India and Australia. Notably, some submitters had identified this species as “*Cli*. *brunneocaperata* J.A. Cooper” or “*Cli*. *quercina* A. Pearson ex Hora”. However, these identifications are incorrect because both taxa differ from *A*. *crenata* in having smooth spores [74,75]. This species can be easily distinguished from its sister species *A*. *benekei* by its whitish pileus and the presence of cheilocystidia [39].

***Allolepista rhodotoides*** (Singer) Z.M. He & Y.D. Xu, comb. nov.

**Fungal names.** FN 573956.

**Basionym.** *Lepista rhodotoides* Singer, in Pegler, *Kew Bull.*, *Addit. Ser.* 9: 98 (1983).

**Notes.** Based on the morphological data of MO547053 from Mushroom Observer [76], the Jamaican collection exhibits a white pileus, pinkish white lamellae, and grows on termite nests, features that are consistent with the protologue of *Lepista rhodotoides* from Martinique [77], supporting the correctness of the identification. Since the morphological features of this species conform well to our circumscription of *Allolepista*, and its phylogenetic position also falls within this genus (Figure 1), we here propose the new combination *Allolepista rhodotoides*.

**Figure 7:**
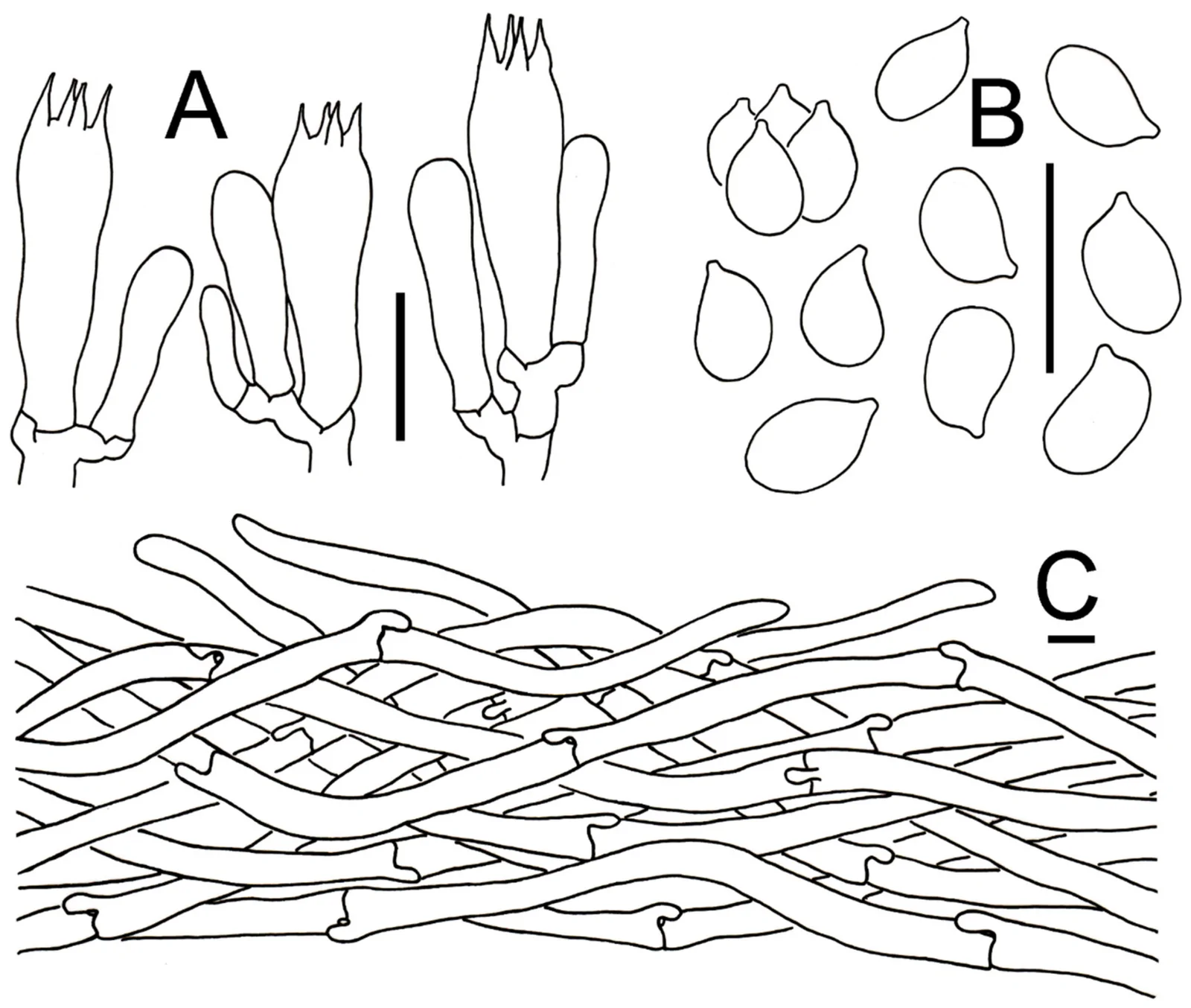
Microscopic features of *Clitocybe sinensis* (MHHNU 32379, holotype). (**A**) Basidia; (**B**) Basidiospores; (**C**) Pileipellis. Bars = 10 μm.

***Clitocybe sinensis*** Z.M. He & Y.D. Xu, sp. nov. (Figure 4C,D and Figure 7).

**Fungal name.** FN 573722.

**Diagnosis.** Similar to *Col*. *humida*, but differs in having a smaller pileus, wider basidiospores, and an interwoven pileipellis.

**Etymology.** “*sinensis*” (Lat.), referring to its exclusive distribution in China.

**Type.** China, Hunan Province, Sangzhi County, Tianping Mountain, on leaf litter, in a coniferous and broad-leaved mixed forest, at 29.35728° N, 110.54367° E, alt. 1455 m, 5 September 2020, Z.H. Chen, MHHNU 32379.

**Description.** Basidiomes small, clitocyboid. Pileus 10–30 mm in diam, at first applanate, then concave; surface smooth, hygrophanous, pale yellow-brown (3A2) to pale gray-brown (4A2) when wet; margin not striate, straight to reflexed, with white (1A1) pruina; context thin (1–2 mm thick), white (1A1). Lamellae adnate to decurrent, crowded, white (1A1) to cream (2A2), arcuate, narrow (about 2 mm high); edges even. Stipe 15–40 × 2–6 mm, central, equal, hollow; surface whitish (2A1), longitudinally fibrillose to slightly tomentose; base finely whitish (2A1) tomentose.

Basidiospores [60/3/3] 5–6 (6.5) × 4–5(5.5) μm, Q = (1.02)1.08–1.51(1.59), Qm = 1.28 ± 0.13, broadly ellipsoid to ellipsoid, smooth, hyaline, thin-walled, inamyloid, weakly cyanophilic, often in tetrads. Basidia 18–25 × 6–7.5 μm, clavate, four-spored, hyaline, thin-walled; sterigmata up to 4 μm long. Cystidia absent. Lamella trama regular; hyphae 3–10 μm wide, cylindrical, hyaline, thin- to slightly thick-walled (<0.5 μm). Pileipellis a cutis composed of subparallel to interwoven, thin- to slightly thick-walled (<0.5 μm), cylindrical hyphae 2–9 μm wide, in places with irregularly raised hyphal ends. Stipitipellis a cutis composed of parallel to subparallel, thin-walled, cylindrical hyphae 3–8 μm wide. Clamp connections present in all parts of the basidiome.

**Ecology.** Usually in clusters of 2–3 basidiomes, on leaf litter, in temperate and subtropical forests; autumn.

**Distribution.** Known from Central and Northwestern China.

**Additional specimens examined.** China, Hunan Province, Sangzhi County, Tianping Mountain, on broad leaf litter, at 29.35928° N, 110.54207° E, alt. 1455 m, 7 September 2020, Z.H. Chen MHHNU 34600; Ningxia Hui Autonomous Region, Yanchi County, on soil, in a broad-leaved forest, at 37.77785° N, 107.40906° E, alt. 1341 m, 17 October 2025, F. Xu, P3161.

**Notes.** The six-locus phylogenetic analysis shows that *Cli. sinensis* clusters together with *Col*. *alboclitocyboides* Z.M. He & Zhu L. Yang, *Col*. *asiatica* Z.M. He & Zhu L. Yang, *Col*. *humida*, *Col*. *phyllophila* (Pers.) Z.M. He & Zhu L. Yang, and *Col*. *tomentostipes* Z.M. He & Zhu L. Yang, forming a clade (66% BP, 0.98 PP; Figure 2). *Collybia alboclitocyboides*, *Col*. *humida*, and *Col*. *phyllophila* can be distinguished from *Cli*. *sinensis* by their larger pileus and narrower basidiospores [12,35]. *Collybia asiatica* differs from *Cli*. *sinensis* by narrower basidiospores and a regular pileipellis, while *Col*. *tomentostipes* differs by an incurved to inrolled pileus margin and narrower basidiospores [12].

The specimen P3161 of *Cli*. *sinensis* was obtained from a case of mushroom poisoning involving two individuals presenting with muscarinic syndrome [42], indicating that *Cli*. *sinensis* should be considered a muscarine-containing species.

**Figure 8:**
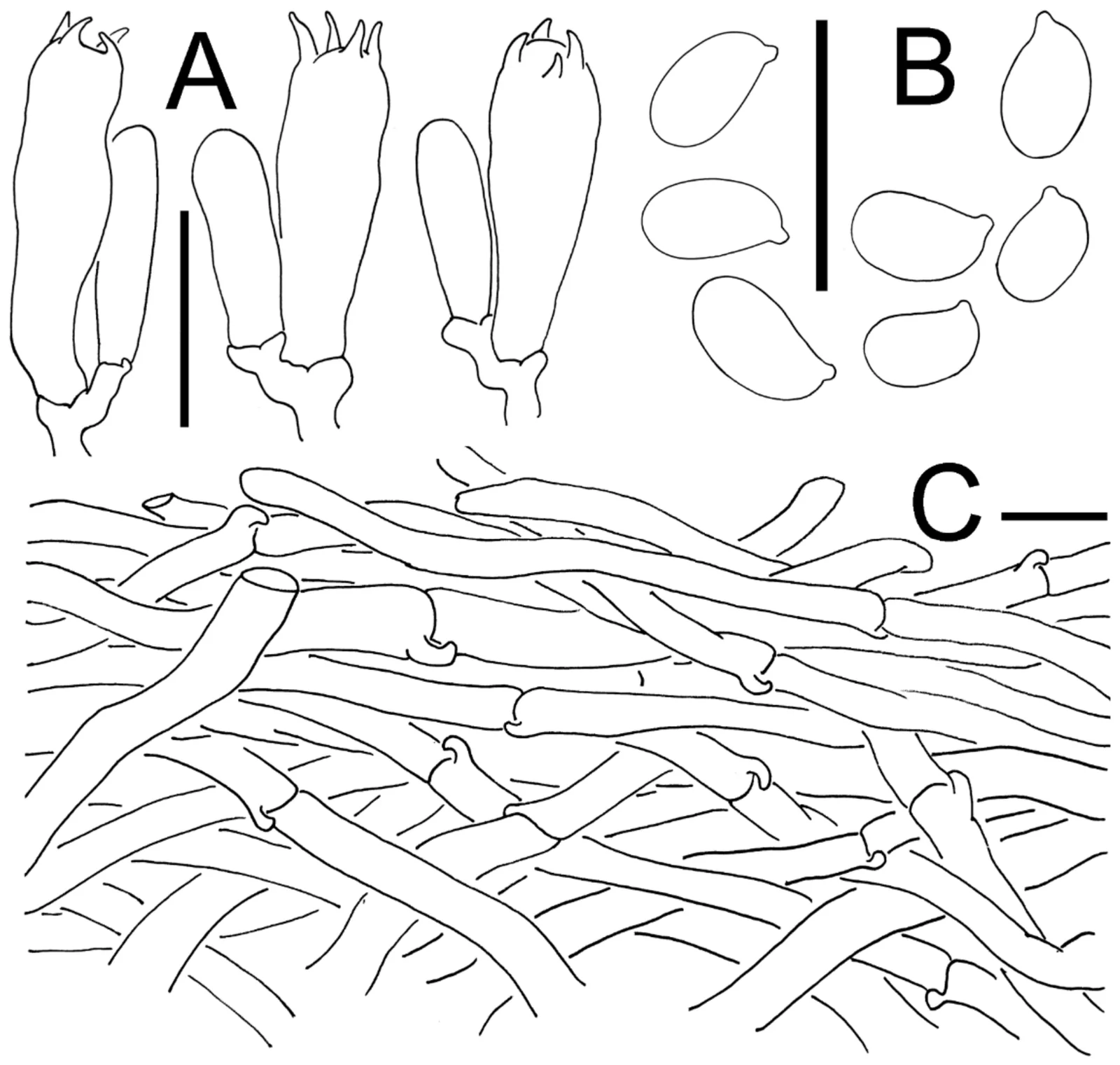
Microscopic features of *Clitocybe truncicola* (MHHNU 20081). (**A**) Basidia; (**B**) Basidiospores; (**C**) Pileipellis. Bars = 10 μm.

***Clitocybe truncicola*** (Peck) Sacc. Sylloge Fungorum (Abellini) V: 184 (1887) (Figure 4E–G and Figure 8).

**Basionym.** *Agaricus truncicola* Peck, *Bull. Buffalo Soc. nat. Sci.* 1(2): 46 (1873) [1873–1874].

= *Clitocybe lamoureae* Armada, Bellanger, Bidaud & P.-A. Moreau, in Armada, Bellanger, Bidaud & Moreau, *Bull. Mycol. Bot. Dauphiné Savoie* 251: 59 (2023).

= *Collybia urbana* L. Fan & Y.X. Zhang, in Zhang, Mao, Fan, Yang, Fan, *Mycologia* 118 (2): 433 (2026).

**Description.** Basidiomes small to medium-sized, clitocyboid. Pileus 30–50 mm in diam, at first convex, later plano-convex to applanate; surface nearly smooth, strongly white-pruinose (1A1), whitish (2A1) to buff (2A3), hygrophanous; center depressed with age; margin not striate, at first inrolled, then expanding to straight, becoming broadly undulate in age; context thin (1–3 mm thick), white (1A1) when dry, cream (2A2) to yellowish (1A2) when wet. Lamellae (sub)decurrent, crowded, whitish (2A1) to cream (2A2), narrow (about 3 mm high); edges even. Stipes 25–50 × 4–10 mm, eccentric or central, equal, sometimes slightly curved, stuffed or hollow; surface nearly smooth, buff (1A2–3), with finely white (1A1) fibrils longitudinally; base covered with sparse white (1A1) mycelium; context concolorous with stipe surface, watery.

Basidiospores [60/6/6] (3)4–6 × (2)2.5–4 μm, Q = (1.14)1.25–1.80(1.96), Qm = 1.53 ± 0.17, broadly ellipsoid to ellipsoid, occasionally elongate, smooth, hyaline, thin-walled, inamyloid, weakly cyanophilic, always single. Basidia 12–20 × 4–6 μm, clavate, four-spored, hyaline, thin-walled; sterigmata up to 3 μm long. Cystidia absent. Lamella trama regular to subregular; hyphae 2–5(6) μm wide, hyaline, cylindrical, thin-walled. Pileipellis a cuits composed of subparallel to interwoven, thin-walled, cylindrical hyphae 3–10(12) μm wide, with exserted ends. Stipitipellis a cutis composed of thin-walled cylindrical hyphae 4–8 μm wide. Clamp connections present in all parts of the basidiome.

**Ecology.** Usually scattered or gregarious, rarely solitary, on rotting wood or humus; autumn.

**Distribution.** Asia, Europe, and North America.

**Specimens examined.** China, Guizhou Province, Baiyun District, Penglai Village, on humus, at 26.74327° N, 106.73246° E, 30 November 2019, alt. 1275 m, Z.H. Chen, MHHNU 31841; Guiyang City, on the ground under trees of *Pinus* sp., 5 October 2022, H.J. Li, MHHNU 33804; Heilongjiang Province, Dongan District, Mudanfeng National Nature Reserve, on hardwood logs, at 44.46685° N, 129.73603° E, alt. 536 m, 15 August 2023, C.G. Sheng, MHHNU 20081; Ningxia Hui Autonomous Region, Jingyuan County, Baitong Road, on needle litter, at 35.39912° N, 106.36842° E, alt. 1799 m, 13 September 2024, Z.H. Chen, MHHNU 34609; Jingyuan County, Zhijia Village, on needle leaf litter, at 35.40071° N, 106.37212° E, alt. 1816 m, 16 September 2024, Z.H. Chen, MHHNU 34699; Yunnan Province, Kunming City, Kunming Botanical Garden, on humus under trees of *Cupressus torulosa* D. Don., at 24.948242° N, 102.749744° E, alt. 1990 m, 24 September 2022, Z.L. Yang, KUN-HKAS 125840.

**Notes.** *Clitocybe truncicola* was first described from the USA by Peck [78], who reported that it has a white, expanded or centrally depressed pileus, adnate-decurrent lamellae, and grows on tree trunks. This species was later described in more detail by Bigelow [35]. It has also been reported in the UK and China [79,80]. The morphological and ecological features of the Chinese specimens examined in this study (Figure 4E and Figure 8) conform well to those descriptions. The size of basidiospores of the Chinese specimens we measured [(3)4–6 × (2)2.5–4 μm] is also comparable to that reported in monographs (Bigelow [35]: 3.5–4.5(5) × 2.5–3.5(4) μm; Buczacki et al. [79]: 4.5–5.5 × 3.5–4 μm). Given these lines of evidence, these Chinese specimens are identified as *Cli. truncicola*.

The phylogenetic analyses (Figure 1 and Figure 2) show that the sequences we identified as *Cli. truncicola* cluster with the sequences of the holotypes of *Cli*. *lamoureae* and *Col*. urbana with very low genetic differences, indicating that these sequences are conspecific. After comparing the descriptions of *Cli*. *lamoureae* [81] and *Col*. *urbana* [41] with the original descriptions and figures of *Cli. truncicola* in Peck [78] and Bigelow [35], we found that these species are morphologically consistent, except that *Cli*. *lamoureae* and *Col*. *urbana* were reported on soil with leaf litter. However, this difference in substrate cannot be regarded as a specific characteristic, since some species of the *Collybia* clade in Clitocybaceae are known to be both terrestrial and lignicolous, e.g., *Col*. *humida* [12] and *Cli*. *violaceifolia* [68]. Armada et al. [81] observed only one specimen of their species, and Zhang et al. [41] observed multiple specimens of their species at the same locality. Consequently, they may have overlooked the possibility of lignicolous individuals. In contrast, our extensive field observations show that *Cli. truncicola* is found on both soil and wood. Therefore, we conclude that *Cli*. *lamoureae* and *Col*. *urbana* are synonyms of *Cli*. *truncicola*.

The specimen MHHNU 33804 of *Cli*. *truncicola* was obtained from a mushroom poisoning incident in Guizhou Province, China, in 2022, affecting seven patients who developed muscarinic syndrome [25]. This species was also responsible for another incident in this province in 2025 [42].

**Figure 9:**
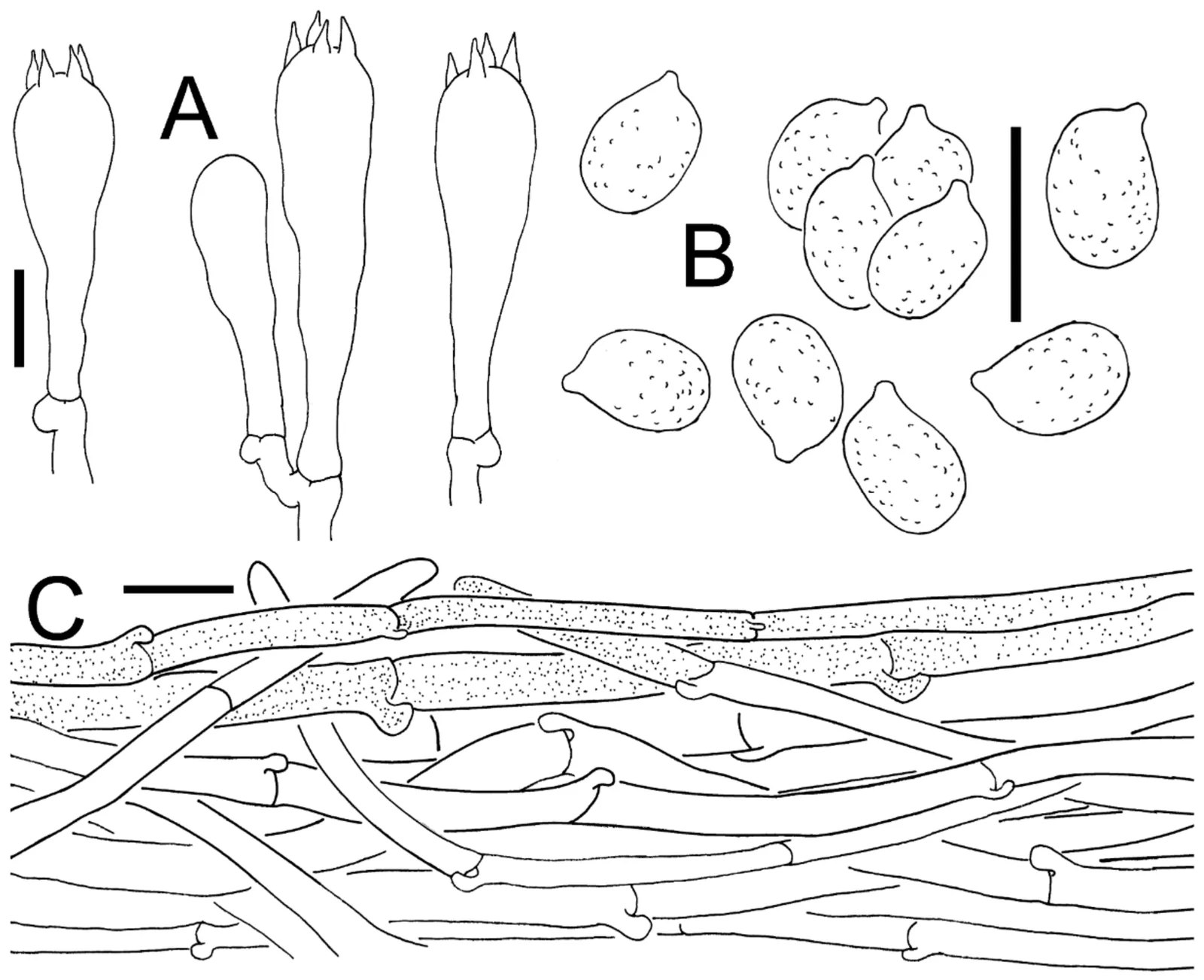
Microscopic features of *Clitocybe violaceifoila* (MHHNU 33773). (**A**) Basidia; (**B**) Basidiospores; (**C**) Pileipellis. Bars = 10 μm.

***Clitocybe violaceifolia*** Murrill, *Mycologia* 5: 213 (1913) (Figure 4H, Figure 5C,D and Figure 9).

**Description.** Basidiomes medium-sized, clitocyboid to tricholomoid. Pileus about 35 mm in diam, convex to hemispherical; surface smooth, glabrous, gray-brown (1B2) with a grayish violet tinge (5B2), not hygrophanous; margin not striate, inflexed to involute; context 8–10 mm thick, white (1A1). Lamellae sinuate to emarginate, crowded, arcuate, grayish (1B1) to pale violet (13B2), 3–5 mm high; edge even. Stipe 30–45 × 6–8 mm, central, equal, slightly enlarged towards the base, solid; surface concolorous with pileus or paler, longitudinally white- to grayish-fibrillose (1A1/1B1); base covered with abundant whitish (2A1) tomentum.

Basidiospores [60/1/1] (5.5)6–8 × 4–5(5.5) μm, Q = (1.18)1.30–1.75(1.88), Qm = 1.48 ± 0.14, (broadly) ellipsoid, thin-walled, inamyloid, cyanophilic, often adhering in tetrads, with numerous, minute, low (<0.2 μm high) verrucae (undimpled apically under SEM). Basidia 25–45 × 7–10 μm, clavate, four-spored, hyaline, thin-walled; sterigmata up to 5 μm long. Cysidia absent. Lamella trama regular to subregular; hyphae 5–10 μm wide, cylindrical, thin-walled. Pileipellis a cutis composed of parallel to slightly interwoven, thin-walled, cylindrical hyphae 5–15 μm wide, with brownish (1B2) to grayish (1B1) intracellular pigment. Stipitipellis a cutis, composed of thin- to slightly thick-walled (<0.5 μm), cylindrical hyphae 3–10(12) μm wide. Clamp connections present in all parts of the basidiome.

**Ecology.** Usually solitary or scattered, on rich soil among herbs or mosses, also on decaying wood; autumn and winter.

**Specimen examined.** China, Inner Mongolia Autonomous Region, Alashanzuo County, Helan Mountain National Nature Reserve, on humus, at 38.34722° N, 105.69833° E, alt. 2471 m, 13 September 2022, Z.H. Chen, MHHNU 33773.

**Distribution.** Known from Northwestern China and Western United States.

**Notes.** The Chinese specimen MHHNU 33773 (Figure 4H) has a gray-brown convex pileus, slightly decurrent grayish violet lamellae, a fleshy context, a solid thick stipe, and ellipsoid spores measuring 6–8 × 4–5 μm. These features correspond well to Murrill’s [82] original description of *Cli*. *violaceifolia* from the Pacific coast of the USA (spores: 7–8 × 3.5–4.5 μm). In the ITS phylogenetic tree (Figure 1), the Chinese specimen clusters with the holotype of *Cli*. *violaceifolia* (OR886357). The ITS sequence of the Chinese specimen shows 99.5% pairwise identity with that of the holotype, indicating that they are conspecific. This morphological and molecular evidence suggests that the Chinese specimen should be identified as *Cli*. *violaceifolia*.

Notably, the Chinese *Cli*. *violaceifolia* were found on soil, whereas Murrill [82] first discovered this species on decaying wood. This implies that this species is both lignicolous and terricolous. It should also be noted that Murrill [82] described it as having smooth spores. However, the spores of the Chinese specimen are finely verrucose under a light microscope (Figure 9B), a feature further confirmed by SEM (Figure 5C,D). The six-locus phylogenetic analysis reveals that *Cli*. *violaceifolia* falls within *Col*. subgen. *Leucocalocybe* (Clade A2; Figure 2). At present, all other known species of this clade are known to produce rough spores (Figure 2) [35,38,40,41]. Hence, it is reasonable that *Cli*. *violaceifolia* possesses verrucose spores.

**Figure 10:**
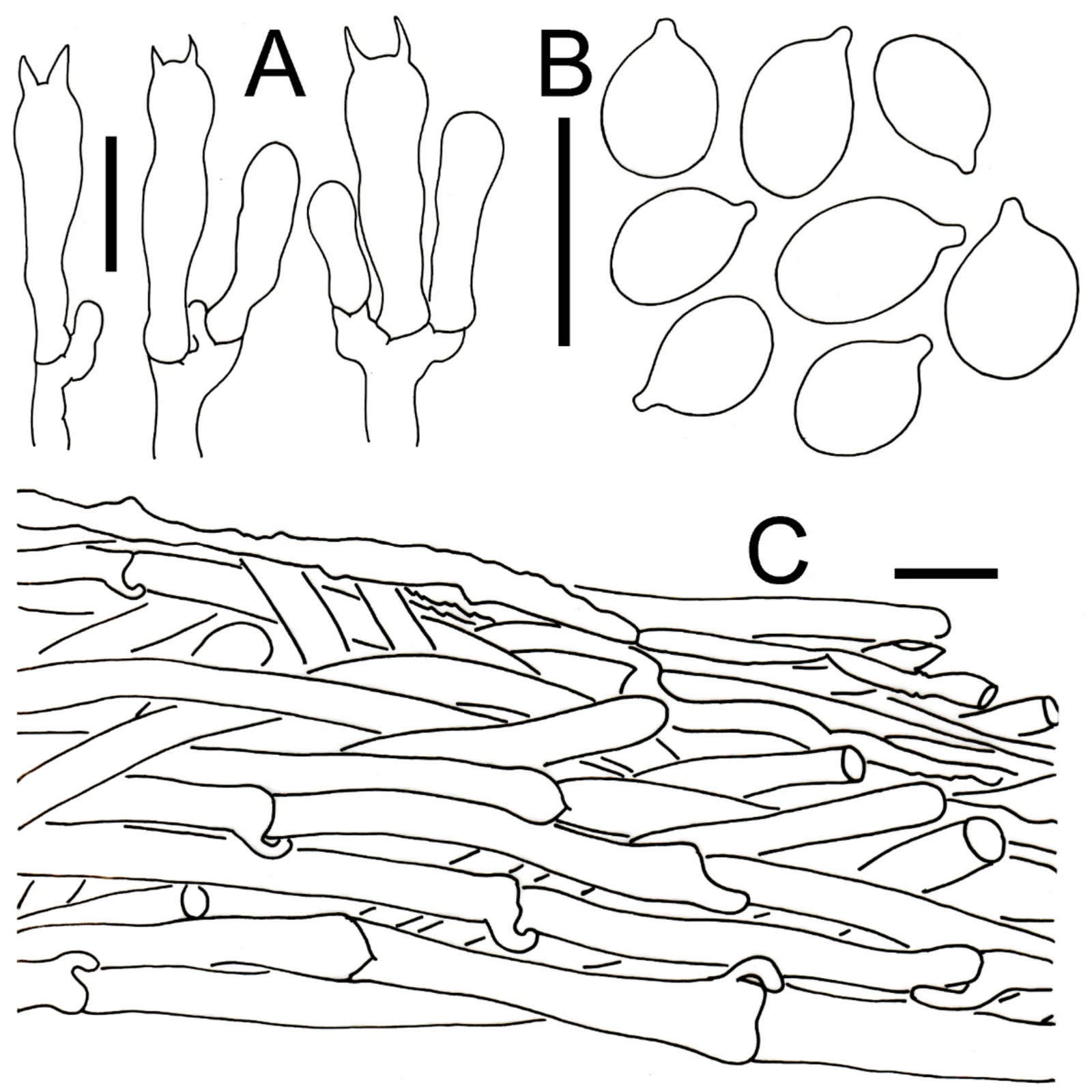
Microscopic features of *Collybia bisterigmata* (MHHNU 33805). (**A**) Basidia; (**B**) Basidiospores; (**C**) Pileipellis. Bars = 10 μm.

***Collybia bisterigmata*** Z.M. He & Zhu L. Yang, in He, Chen, Bau, Wang & Yang, *Fungal Diversity* 123: 21 (2023) (Figure 4I and Figure 10).

**Description.** Basidiomes small, clitocyboid. Pileus 20–30 mm in diam, plano-convex to applanate; surface hygrophanous, off-white (1A2) in dry areas, brownish (4B2) in moist areas; center depressed when the pileus expands; margin with distinct white (1A1) pruina; context 1–2 mm thick, white (1A1). Lamellae (sub)decurrent, crowded, white (1A1), 1–2 mm high; edges even. Stipe 25–30 × 2–3 mm, brownish (4B2), central, equal, hollow, tomentose at base.

Basidiospores [60/2/1] (5)6–7(8) × 4–6 μm, Q = (1.00)1.12–1.48(1.56), Qm = 1.27 ± 0.11, (broadly) ellipsoid, smooth, inamyloid, weakly cyanophilic, always single. Basidia 20–35 × 6–8 μm, clavate, two-spored; sterigmata up to 15 μm long. Cystidia absent. Lamella trama subregular, composed of cylindrical to inflated hyphae 4–25 µm wide. Pileipellis a cutis composed of subparallel cylindrical hyphae 2–10 μm wide. Stipitipellis a cutis composed of cylindrical hyphae 3–10 μm wide. Clamp connections present in all parts of the basidiome.

**Ecology.** Gregarious, on leaf litter, in subtropical forests; autumn.

**Distribution.** Known from Southwestern China.

**Specimen examined.** China, Yunnan Province, Zhenxiong County, Shangjie Village, on fallen leaves of *Cunninghamia lanceolata* (Lamb.) Hook., at 27.44618° N, 104.86974° E, alt. 1700 m, 19 October 2022, H.J. Li, MHHNU 33805.

**Notes.** The specimen MHHNU 33805, identified as *Col*. *bisterigmata*, was collected from a mushroom poisoning incident that occurred in Yunnan Province in 2022 [25]. This incident involved 20 patients whose symptoms were classified as muscarinic syndrome (misidentified as *Col*. *humida* in Li et al. [25]). This outcome matches our previous study [12], in which this species was found to contain muscarine.

**Figure 11:**
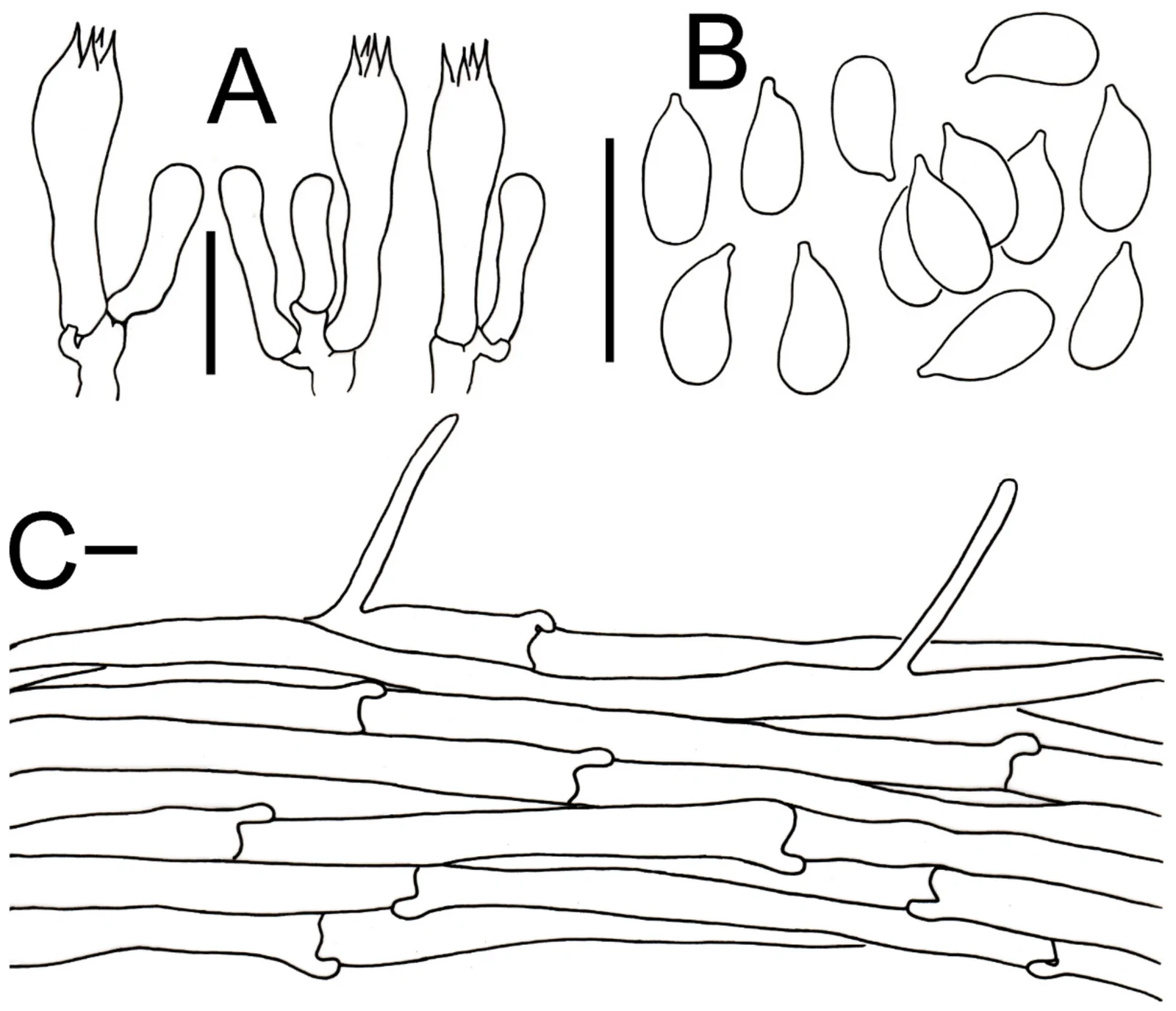
Microscopic features of *Collybia brunneoumbilicata* (MHHNU 20070). (**A**) Basidia; (**B**) Basidiospores; (**C**) Pileipellis. Bars = 10 μm.

***Collybia brunneoumbilicata*** Z.M. He & Zhu L. Yang, in He, Chen, Bau, Wang & Yang, *Fungal Diversity* 123: 22 (2023) (Figure 4J and Figure 11).

**Description.** Basidiomes small, clitocyboid. Pileus about 30 mm in diam, convex to plano-convex with a depressed center; surface pruinose, hygrophanous, white (1A1) when dry, brownish (4B2) when wet; margin usually inflexed, with zonated pruina; context white (1A1), 0.5–2 mm thick. Lamellae decurrent, crowded, whitish (2A1), with a yellowish brown (4A2) tinge, arcuate, 1–2 mm high; edges even. Stipe 10–30 × 1–3 mm, central, equal, solid when young, hollow when mature; surface brownish (3B3), becoming white (1A1) when covered with longitudinal fibrils; base white tomentose (1A1); context white (1A1). Odor strongly fragrant.

Basidiospores [60/5/5] 4–5.5 × 2–3 μm, Q = (1.33)1.59–2.50(2.75), Qm = 1.95 ± 0.29, mostly elongate to cylindrical, smooth, inamyloid, weakly cyanophilic, sometimes in tetrads. Basidia 15–25 × 4–8 μm, clavate, four-spored; sterigmata up to 4 μm long. Pleurocystidia and cheilocystidia absent. Lamella trama regular; hyphae 3–10 µm wide, hyaline, cylindrical, thin- to slightly thick-walled (<0.5 μm). Pileipellis a cutis composed of parallel, thin- to slightly thick-walled (<0.5 μm), cylindrical hyphae 3–10 µm wide, with narrowly cylindrical pileocystidia 18–25 × 2–3 μm. Stipitipellis a cutis composed of parallel, thin- to slightly thick-walled (0.5–1 μm), cylindrical hyphae 2–10 μm wide, with narrowly cylindrical caulocystidia 15–30 × 2–3 μm. Clamp connections present in all parts of the basidiome.

**Ecology.** Gregarious, on soil or leaf litter, in subtropical forests; summer and autumn.

**Distribution.** Known from Central and Southwestern China.

**Specimens examined.** China, Guizhou Province, Renhuai County, Shangzhai Village, on leaf litter, at 27.91537° N, 106.57876° E, alt. 1067 m, 3 November 2023, Z.H. Chen, MHHNU 34172; Renhuai County, Shangzhai Village, on broad leaf litter, at 27.97536° N, 106.57890° E, alt. 1117 m, 3 November 2023, Z.H. Chen, MHHNU 34177; Renhuai County, Shangzhai Village, on broad leaf litter, at 27.91527° N, 106.57878° E, alt. 1169 m, 3 November 2023, Z.H. Chen, MHHNU 34179; Hunan Province, Xuefeng County, Daping Village, on leaf litter of *Cunninghamia lanceolata*, at 27.32577° N, 110.28480° E, alt. 288 m, 3 November 2023, Z.M. He, MHHNU 20068; Xuefeng County, Daping Village, on leaf litter of *Cunninghamia lanceolata*, at 27.32607° N, 110.28410° E, alt. 290 m, 3 November 2023, Z.M. He, MHHNU 20070.

**Notes.** In 2023, two mushroom poisoning incidents involving this species occurred in Central China and Southwestern China, respectively [43]. Our five specimens were collected from the sites of these incidents. In both cases, the patients developed symptoms within 30 min, including nausea, vomiting, diarrhea, shivering, drooling, and dizziness, a typical clinical presentation corresponding to muscarinic syndrome. This outcome is consistent with our previous finding that this species contains muscarine [12]. He et al. [12] only reported *Col*. *brunneoumbilicata* occurring in broad-leaved forests, whereas we additionally found it in coniferous forests.

**Figure 12:**
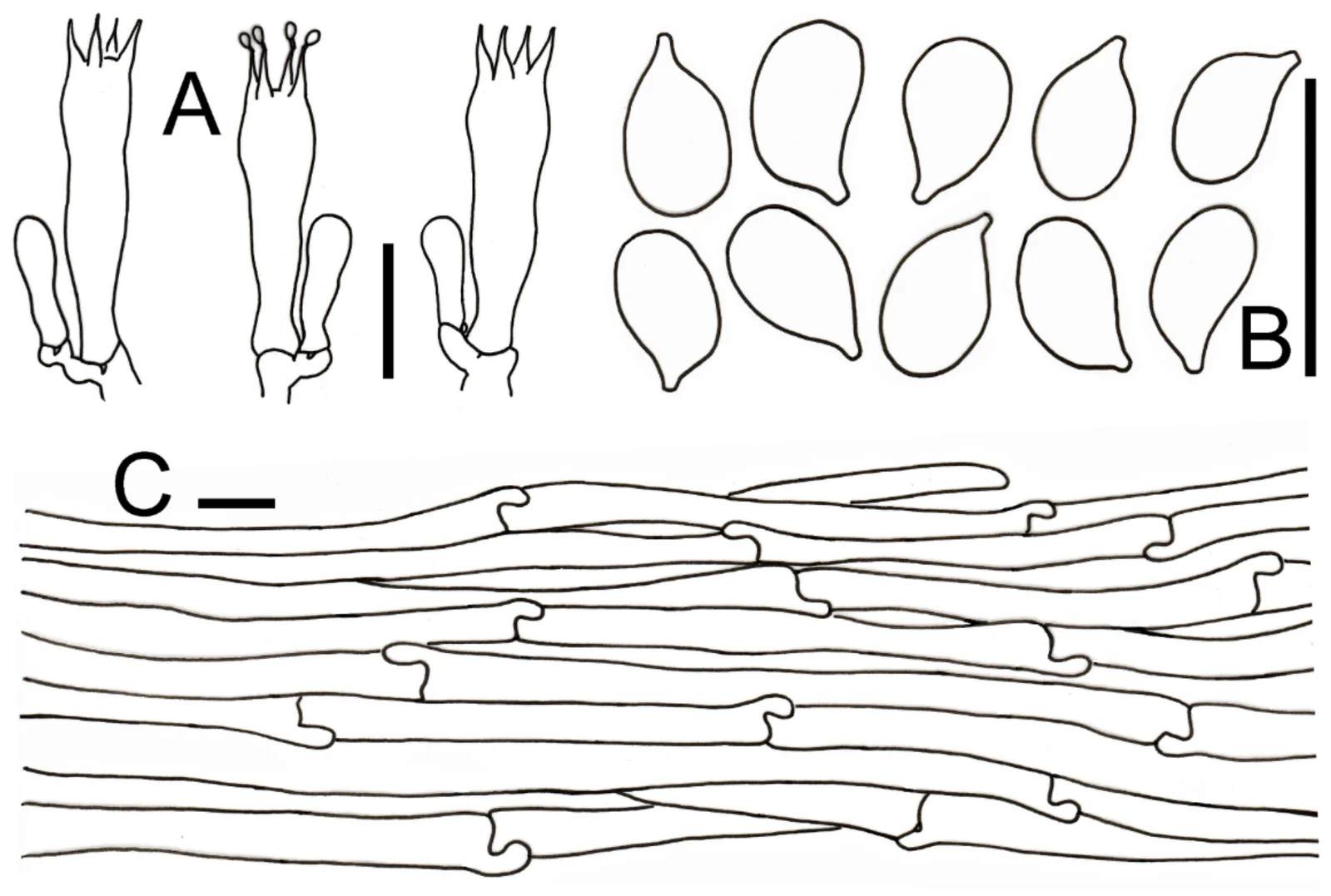
Microscopic features of *Collybia subtropica* (MHHNU 34153). (**A**) Basidia; (**B**) Basidiospores; (**C**) Pileipellis. Bars = 10 μm.

***Collybia subtropica*** Z.M. He & Zhu L. Yang, in He, Chen, Bau, Wang & Yang, *Fungal Diversity* 123: 29 (2023) (Figure 4K and Figure 12).

**Description.** Basidiomes small, clitocyboid. Pileus about 35 mm in diam, convex, applanate with umbilicate at center in maturity; surface smooth, brownish (3A2–3) to pinkish brown (5A2), strongly white-pruinose (1A1), hygrophanous; margin always incurved; context thin (1–2 mm thick), buff (1A2). Lamellae adnate to (sub)decurrent, crowded, cream (2A2), narrow (<2 mm high), sometimes forked; edges even. Stipe 20–50 × 2–6 mm, equal, hollow, finely fibrillose, yellowish brown (5A1–3); base white tomentose (1A1); context brownish (3A2) when wet, becoming whitish (2A1) when dry.

Basidiospores [60/4/4] (4)4.5–6 × (2)2.5–4 μm, Q = (1.15)1.21–2.12(2.45), Qm = 1.64 ± 0.30, ellipsoid to elongate, smooth, inamyloid, weakly cyanophilic, always single. Basidia 20–30 × 4.5–6 μm, clavate, four-spored, hyaline; sterigmata up to 5 μm long. Cystidia absent. Lamella trama regular; hyphae 3–14 µm wide, hyaline, cylindrical, thin-walled. Pileipellis a cutis composed of parallel, thin-walled, cylindrical hyphae 2–8 µm wide. Stipitipellis a cutis composed of cylindrical hyphae 3–10 μm wide. Clamp connections present in all parts of the basidiome.

**Ecology.** Gregarious, on soil or leaf litter; summer and autumn.

**Distribution.** Known from subtropical zones of Central and Southwestern China.

**Specimens examined.** China, Hunan Province, Yongding District, Gaogong Village, on needle and broad leaf litter of *Pinus massoniana* Lamb. and *Castanopsis sclerophylla* (Lindl.) Schottky, at 29.21747° N, 110.57613° E, alt. 350 m, 9 October 2023, Z.M. He, MHHNU 34153; Yongding District, Gaogong Village, on needle and broad leaf litter of *Pinus massoniana* and *Castanopsis sclerophylla*, at 29.21705° N, 110.57621° E, alt. 348 m, 9 October 2023, Z.M. He, MHHNU 34154; Zhongfang County, Zhanping Village, on soil, in a forest of *Acer* sp., at 27.37560° N, 109.92461° E, alt. 203 m, 13 November 2023, Z.H. Chen, MHHNU 34191; Huitong County, 17 November 2023, Z.H. Chen, MHHNU 34194.

**Notes.** Our four specimens (MHHNU 34153, 34154, 34191, and 34194) of *Col. subtropica* were collected in 2023 from the sites of three mushroom poisoning incidents in Hunan Province, China [43]. This species was also responsible for a mushroom poisoning incident in Hunan Province in 2018 [12] and one in Yunnan Province in 2020 [83]. Therefore, it is the primary species causing poisoning within *Collybia* in China. In the wild, the bicolored appearance, consisting of a whitish pileus and a yellowish brown stipe, serves as a diagnostic character for recognizing this species among other small white clitocyboid fungi.

**Figure 13:**
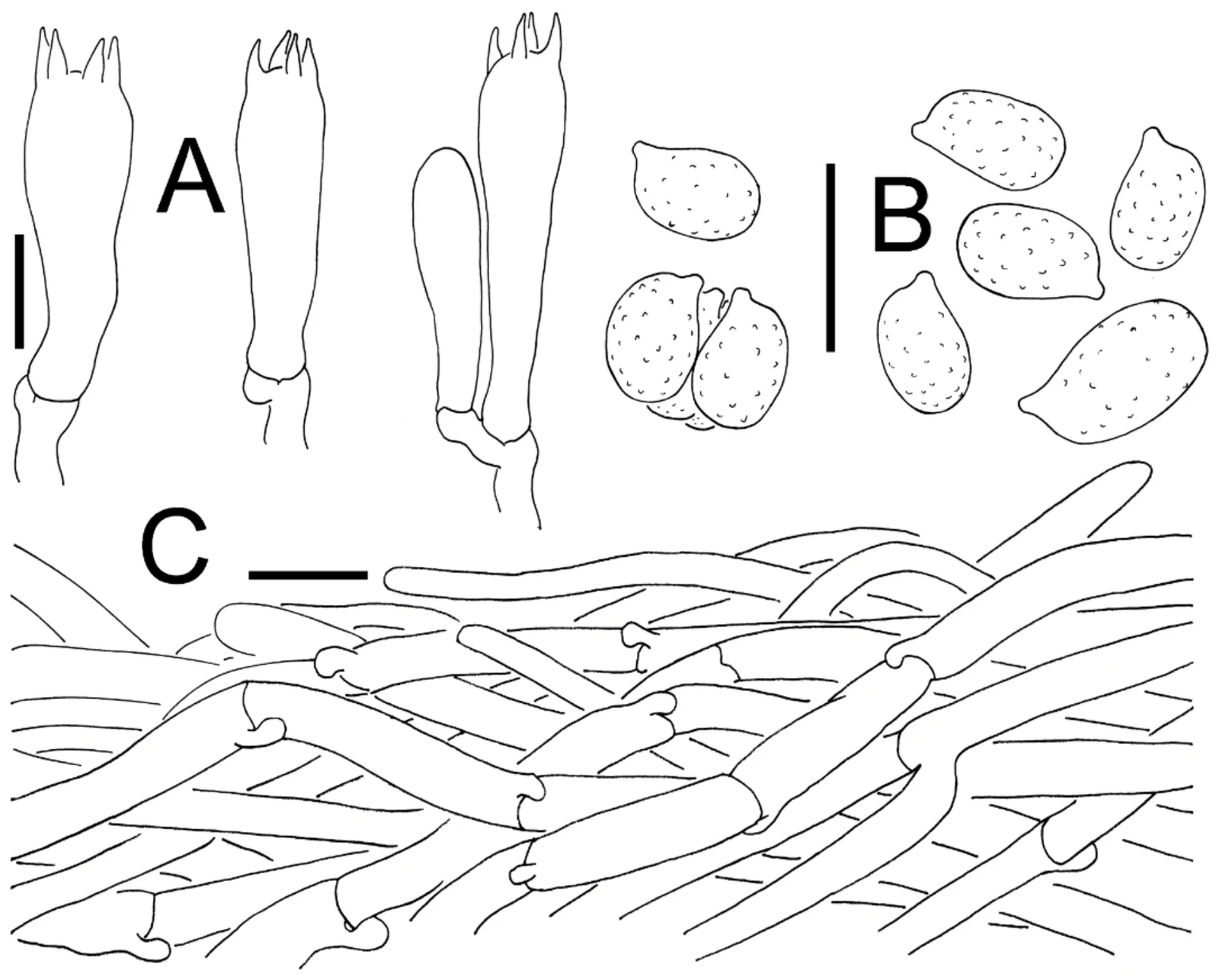
Microscopic features of *Collybia violea* (MHHNU 34607). (**A**) Basidia; (**B**) Basidiospores; (**C**) Pileipellis. Bars = 10 μm.

***Collybia violea*** X.D. Yu & H.B. Guo, in Qi, Xu, Yang, Guo, Guo, Wan, Yang, Pei & Yu, *Journal of Fungi* 11: 6 (2025) (Figure 4L, Figure 5E,F and Figure 13).

**Synonymy.** *Collybia sinoborealis* L. Fan & Y.X. Zhang, in Zhang, Mao, Fan, Yang, Fan, *Mycologia* 118: 433 (2026).

**Description.** Basidiomes small to medium, clitocyboid. Pileus 30–50 mm in diam, at first convex to plano-convex, becoming nearly applanate when mature; surface glabrous, grayish lilac (12B2) to brownish lilac (4B2), with brownish white (1B2) pruinose, slightly hygrophanous; margin not striate, incurved; context thin (<3 mm thick), brownish gray (5A3) to grayish white (5B1), watery. Lamellae sinuate to emarginate, moderately crowded, violet-gray (13B2) when moist, brownish (4B2) with grayish violet tinge (13B3) when dry, broad (<5 mm high); edges even. Stipe 40–50 × 6–10 mm, equal, solid; surface concolorous with pileus, longitudinally whitish-fibrillose (2A1) on grayish violet (11B2) background; base sometimes inflated; context grayish violet (11B2) to pinkish (11A2).

Basidiospores [60/3/2] 6–8 × (3.5)4–5(5.5) μm, Q = (1.20)1.29–1.77(1.88), Qm = 1.54 ± 0.16, ellipsoid to broadly ellipsoid, hyaline, thin-walled, inamyloid, cyanophilic, sometimes adhering in tetrads, with low (<0.25 μm high), crowed verrucae (undimpled apically under SEM). Basidia 23–35 × 6–10 μm, clavate, four-spored, hyaline, thin-walled; sterigmata up to 5 μm long. Cystidia absent. Lamella trama regular to subregular; hyphae 3–10 μm wide, thin-walled, cylindrical. Pileipellis a cutis composed of subparallel, thin-walled, cylindrical hyphae 5–10 μm wide. Stipitipellis a cutis composed of thin-walled cylindrical hyphae 3–15 μm wide. Clamp connections present in all parts of the basidium.

**Ecology.** Gregarious or scattered, on soil; summer and autumn.

**Distribution.** Known from Northern, Northeastern, and Northwestern China.

**Specimens examined.** China. Ningxia Hui Autonomous Region, Longde County, Liupan Mountain, on soil with needle and leaf litter, at 35.61668° N, 106.12019° E, alt. 2700 m, 10 September 2022, Z.H. Chen, MHHNU 33752; Jingyuan County, Baitong Road, on soil, at 35.39910° N, 106.36841° E, alt. 1799 m, 13 September 2024, Z.H. Chen, MHHNU 34607.

**Notes.** The phylogenetic analyses (Figure 1 and Figure 2) show that our specimens MHHNU 33752 and 34607 group together with the holotypes of *Col*. *sinoborealis* (SYAU-FUNGI-052) and *Col*. *violea* (BTJC FM4990), exhibiting extremely low genetic difference. The pairwise identity among the ITS sequences of these specimens ranges from 99.48% to 99.82%. This molecular evidence strongly suggests that these specimens belong to the same species. Moreover, our specimens also align well with the original descriptions of *Col*. *violea* by Qi et al. [40] and *Col*. *sinoborealis* by Zhang et al. [41], except for spore size. The spores of our specimens, measuring 6–8 × 4–5 μm, are slightly larger than those reported in Qi et al. [40] (4.5–7.0 × 3.2–4.0 µm), but are comparable to those in Zhang et al. [41] (6.5–7.5 × 4.5–5.5 μm). This can be considered an intraspecific difference. According to Art. 11.3 of the ICN, the earlier name *Col*. *violea* has priority over *Col*. *sinoborealis*. Hence, we use *Col*. *violea* for our specimens.

## 4. Discussion

### 4.1. Clitocybe as the Genus Name for the New Clitocyboid Species

Our phylogenetic analyses (Figure 1, Figure 2 and Figure 3) are consistent with the findings of the previous study by He et al. [12]. Except for the current type species of *Clitocybe*, *Cli*. *nebularis* (Clade G), most traditional Clitocybe species fall within the *Collybia* clade (Clade A). This clade is further divided into four clades (Clades A1–A4), including species previously classified under (1) *Cli*. sect. *Candicantes* (Quél.) Konrad & Maubl. [84], *Cli*. sect. *Odorae* H.E. Bigelow [35], and *Collybia* s.str. [85]; (2) *Lepista* sect. Nuda Harmaja [86]; (3) *Cli*. sect. *Fragrans* (Harmaja) Singer [36]; and (4) the *Lepista irina* (Fr.) H.E. Bigelow complex.

In view of this situation, He and Yang [11] proposed moving the current type species of *Collybia* (*Col*. *tuberosa*) and its allied species into *Clitocybe*, with *Cli*. *phyllophila* as the new type. This proposal is mainly grounded in their placement within the core muscarine-containing clade, which includes the majority of traditional *Clitocybe* species. Based on shared chemical and molecular evidence, we consider them congeneric. Additionally, for the traditional *Lepista* species that fall within Clade A, their combinations under *Clitocybe* already exist, as Bigelow and Smith [87] and Bigelow [35] treated *Lepista* as a section of *Clitocybe*. Adopting this transfer would avoid the nomenclatural instability that would result from recombining hundreds of *Clitocybe* species. Meanwhile, *Cli*. *nebularis* would be in a new genus if no other old genus name can be applied to it. Therefore, the present study uses *Clitocybe* as the genus name for the new Chinese species within the *Collybia* clade, viz., *Clitocybe sinensis* (Figure 2), even though the final decision regarding the proposal will be made at the next ICN Congress. Such a naming convention was adopted in a previous study by Martínez et al. [38], viz., *Clitocybe cedrelae*.

### 4.2. Spore Evolution in Clitocybaceae

In Clitocybaceae, most species produce smooth spores, whereas rough spores are found in lepistoid species. By integrating phylogenetic analysis and spore surface data, we found that these rough-spored lepistoid species are clustered into four distinct clades within Clitocybaceae (Figure 2): *Col*. subgen. *Leucocalocybe* (A2), *Col*. subgen. *Crassicybe* (A4), *Lepista* s.str. (C), and *Allolepista* (D). The observation that these clades are not recovered as a single lineage is consistent with multiple independent origins of rough spores in this family.

These independent origins are manifested in the shape of verrucae. Phylogenetic distribution of rough spores (Figure 2) reveals that *Col*. subgen. *Leucocalocybe* (A2) produces spores with low hemispherical verrucae, as observed under SEM in *Cli*. *cedrelae* [38], *Cli*. *violaceifolia* (Figure 5C,D), *Col*. *mongolica* (S. Imai) Z.M. He & Zhu L. Yang [88], *Col*. *nuda*, *Col*. *personata* (Fr.) Z.M. He & Zhu L. Yang, *Col*. *sordida* [89], *Col*. *rhodoviolacea* L. Fan & Y.X. Zhang [41], and *Col*. *violea* [40], etc. *Collbia* subgen. *Crassicybe* (A4) contains only *Col*. *irina*, which produces either smooth spores or spores with low hemispherical verrucae [69]. *Lepista* s.str. (C) produces spores with low hemispherical verrucae, such as *L*. *multiformis* (Schaeff.) Gulden [90], but also produces distinctly hemispherical verrucose spores, such as *L*. *cremeoinvoluta* Xu et al. [21], *L*. *densifolia*, *L*. *subconnexa* (Murrill) Harmaja [69], and *L*. *panaeola* (Fr.) P. Karst. [91]. In contrast to Clades A2, A4, and C, the spore verrucae of *Allolepista* (D) are more strongly developed, presenting an elongated hemispherical shape (Figure 5A,B) [39].

Another piece of evidence supporting independent spore evolution in these four clades is the presence or absence of collapse at the apex of verrucae (i.e., dimpled verrucae). Our SEM observations (Figure 5G,H) show that *L*. *panaeola* possesses spores with distinctly dimpled verrucae. This dimpled feature has also been observed in other known species of this clade (Clade C; Figure 2), e.g., *L*. *cremeoinvoluta* and *L*. *multiformis* [21], indicating that *Lepista* s.str. typically produces dimpled spore verrucae. As for *Col*. subgen. *Leucocalocybe* (A2), this clade is further resolved into two subclades. Species of the first subclade, e.g., *Cli*. *cedrelae*, *Col*. *nuda*, *Col*. *rhodoviolacea*, *Col*. *sordida*, and *Col*. *personata*, also show spores with apical dimples in previous SEM observations [38,41,89]. However, species of the second subclade, e.g., *Cli*. *violaceifolia* (Figure 5C,D) and *Col*. *violea* (Figure 5E,F), lack such dimples on their spore verrucae. Similarly, no apically dimpled spore verrucae have been observed in *Col*. subgen. *Crassicybe* [69,87,89] and *Allolepista* (Figure 5A,B). Consequently, dimpled verrucae are found only in *Lepista* s.str. and the first subclade of *Col*. subgen. *Leucocalocybe*. This polyphyletic distribution, in which even within the same subgenus (A2), one subclade possesses dimpled verrucae while the other one does not, indicates that this feature is not a synapomorphic trait. Instead, this pattern could be interpreted as a case of convergent evolution of spore ornamentation across distantly related lineages within Clitocybaceae, but this interpretation awaits further SEM observations from a broader sampling of species.

### 4.3. Poisonous Species and Toxicities of Clitocybaceae

In the present work, we identified *Cli*. *sinensis*, *Cli*. *truncicola*, *Col*. *bisterigmata*, *Col*. *brunneoumbilicata*, and *Col*. *subtropica*, which have caused muscarinic syndrome in mushroom poisoning incidents in China in recent years. Among these species, the latter four have been confirmed to contain muscarine in previous work [12], while *Cli*. *sinensis* have not. According to our six-locus phylogenetic analysis, *Cli*. *sinensis* falls within *Col*. subgen. *Collybia* (Clade A1; Figure 2). This correlates with our previous finding that muscarine-producing species are restricted to subgen. *Collybia* in Clitocybaceae, with the exception of the earliest diverging species in this subgenus, *Col*. *odora* (Bull.) Z.M. He & Zhu L. Yang, which does not produce muscarine [12].

The other clinical type of poisoning caused by Clitocybaceae is the gastrointestinal syndrome, which has been relatively infrequently documented for this family. To our knowledge, only *L*. *cremeoinvoluta* has been reported to cause this type of poisoning [92]. In this study, we report that the species *A*. *benekei* also causes this syndrome. This correlates with the previous work of Nussbaum [72], who found that administration of undiluted crude extracts from cultivated basidiomes of this species to mice resulted in toxic side effects.

## Figures and Tables

**Table 1:** Sequences used for the six-locus phylogenetic analysis of Clitocybaceae.

Species	Voucher	Locality	ITS	LSU	*TEF1*	*RPB1*	*RPB2*	*ATP6*
***A*. *benekei***	**P2402 🕱**	**China: Guizhou**	**PZ159354**	**PZ176954**	**PZ173014**	–	**PZ173008**	**PZ189223**
***A*. *benekei***	**MHHNU 20080 🕱**	**China: Guizhou**	**PZ159355**	**PZ176955**	**PZ173015**	–	**PZ173009**	**PZ189224**
*A*. *crenata*	UEH-F40069	Pakistan	PX435344	–	–	–	–	–
*A*. *crenata*	UEH-F40070 (T)	Pakistan	PX435343	PX435345	–	–	–	–
*Cli. cedrelae*	BAFC53464 (T)	Argentina	OR886658	OR886661	–	–	–	–
*Cli*. *dealbata*	IE BSG HC95cp3	–	AF357061	AF223175	EF421080	DQ825414	DQ825407	–
*Cli*. *epixyla*	KUN-HKAS 105576 (T)	China: Gansu	OP626916	OP646342	OP558039	OP709952	OP899566	OP956583
*Cli*. *lamoureae*	LY-FA4312 (T)	France	OR419848	–	–	–	–	–
*Cli*. *lamoureae*	Mushroom Observer 284722	USA	MT677863	–	–	–	–	–
*Cli*. *nebularis*	KUN-HKAS 105556	China: Gansu	OP626917	OP646343	OP558040	OP709953	OP899604	OP956584
***Cli*. *sinensis***	**MHHNU 32379 (T)**	**China: Hunan**	**PZ159356**	**PZ176956**	**PZ173016**	–	**PZ173010**	**PZ189225**
***Cli*. *sinensis***	**MHHNU 34600**	**China: Hunan**	**PZ159357**	**PZ176957**	**PZ173017**	–	**PZ173011**	**PZ189226**
***Cli*. *sinensis***	**P3161 🕱**	**China: Ningxia**	**PZ159358**	–	–	–	–	–
*Cli*. *tomentosa*	KUN-HKAS 123097 (T)	China: Yunnan	OP626919	OP646345	–	–	–	–
*Cli*. *tomentosa*	KUN-HKAS 123098	China: Yunnan	OP626920	OP646346	OP558043	OP709955	OP899567	OP956586
*Cli*. *truncicola*	KUN-HKAS 106644	China: Sichuan	OP626970	OP646396	OP672224	OP871378	OP939950	OP956628
***Cli*. *truncicola***	**KUN-HKAS 125840**	**China: Yunnan**	**PZ159359**	**PZ176958**	**PZ173018**	**PZ172997**	–	**PZ189227**
***Cli*. *truncicola***	**MHHNU 20081 🕱**	**China: Heilongjiang**	**PZ159360**	–	–	–	–	–
***Cli*. *truncicola***	**MHHNU 31841**	**China: Guizhou**	**PZ159361**	**PZ176959**	**PZ173019**	**PZ172998**	**PZ212603**	**PZ189228**
***Cli*. *truncicola***	**MHHNU 33804 🕱**	**China: Guizhou**	**PZ159362**	**PZ176960**	**PZ173020**	**PZ172999**	**PZ212604**	**PZ189229**
***Cli*. *truncicola***	**MHHNU 34609**	**China: Ningxia**	**PZ159363**	**PZ176961**	**PZ173021**	**PZ173000**	**PZ212605**	**PZ189230**
***Cli*. *truncicola***	**MHHNU 34699**	**China: Ningxia**	**PZ159364**	**PZ176962**	**PZ173022**	**PZ173001**	**PZ212606**	**PZ189231**
***Cli*. *violaceifolia***	**MHHNU 33773**	**China: Ningxia**	**PZ159365**	**PZ176963**	**PZ173023**	**PZ173002**		**PZ189232**
*Cli*. *violaceifolia*	NY-657675 (T)	USA	OR886357	–	–	–	–	–
*Col*. *alboclitocyboides*	KUN-HKAS 123099 (T)	China: Yunnan	OP626923	OP646349	OP558046	OP763397	OP899608	OP956589
*Col*. *asiatica*	KUN-HKAS 114150 (T)	China: Yunnan	OP626968	OP646394	OP672226	OP871376	OP899584	OP956608
*Col*. *bisterigmata*	KUN-HKAS 123102 (T)	China: Tibet	OP626927	OP646353	OP558050	OP763401	OP899570	OP956593
***Col*. *bisterigmata***	**MHHNU 33805 🕱**	**China: Hunan**	**PZ159366**	**PZ176964**	**PZ173024**	**PZ173003**	**PZ212607**	–
*Col*. *brunneoumbilicata*	KUN-HKAS 108402 (T)	China: Yunnan	OP626928	OP646354	OP558051	OP763402	OP899571	OP956594
***Col*. *brunneoumbilicata***	**MHHNU 20068 🕱**	**China: Hunan**	**PZ159367**	–	**PZ173025**	**PZ173004**	**PZ212608**	–
***Col*. *brunneoumbilicata***	**MHHNU 20070 🕱**	**China: Hunan**	**PZ159368**	–	**PZ173026**	**PZ173005**	**PZ212609**	–
***Col*. *brunneoumbilicata***	**MHHNU 34172 🕱**	**China: Guizhou**	**PZ159369**	**PZ176965**	–	–	–	–
***Col*. *brunneoumbilicata***	**MHHNU 34177 🕱**	**China: Guizhou**	**PZ159370**	**PZ176966**	–	–	–	–
***Col*. *brunneoumbilicata***	**MHHNU 34179 🕱**	**China: Guizhou**	**PZ159371**	**PZ176967**	–	–	–	–
*Col*. *carnea*	SYAU-FUNGI-089 (T)	China: Tibet	PQ594919	PQ594908	PQ609675	–	PQ609669	–
*Col*. *cirrhata*	HMJAU 44754	China: Jilin	OP626931	OP646357	OP558054	OP763405	OP899574	OP956597
*Col*. *clavipes*	SYAU-FUNGI-088 (T)	China: Tibet	PQ594917	PQ594906	PQ801140	–	PQ801138	–
*Col*. *dryadicola*	KUN-HKAS 116076	China: Tibet	OP626934	OP646360	OP558057	OP763408	OP899577	OP956600
*Col*. *humida*	KUN-HKAS 123107 (T)	China: Yunnan	OP626939	OP646365	OP558062	OP763413	OP899582	OP956605
*Col*. *hunanensis*	MHHNU 31389 (T)	China: Hunan	OP626969	OP646395	OP672225	OP871377	OP939979	OP956639
*Col*. *nuda*	KUN-HKAS 93478	China: Heilongjiang	OP626948	OP646374	–	–	–	–
*Col*. *nuda*	11CA041Z	USA	–	KJ021705	–	–	KJ136110	–
*Col*. *odora*	KUN-HKAS 123110	China: Jilin	OP626950	OP646376	OP558069	OP763420	OP899589	OP956612
*Col*. *pannosa*	KUN-HKAS 115428 (T)	China: Tibet	OP626955	OP646381	OP610556	OP763425	OP899593	OP956617
*Col*. *personata*	ADW0097	USA	KJ137270	KJ417193	–	KU139032	KJ424376	–
*Col*. *petaloidea*	KUN-HKAS 101063 (T)	China: Sichuan	OP626956	OP646382	OP610557	OP763426	OP899594	OP956618
*Col*. *phyllophila*	KUN-HKAS 123116	China: Yunnan	OP626964	OP646390	OP672230	OP871372	OP899602	OP956624
*Col*. *piceata*	KUN-HKAS 123117 (T)	China: Xinjiang	OP626965	OP646391	OP672229	OP871373	OP899603	OP956625
*Col*. *rhodoviolacea*	BJTC FM4014 (T)	China: Shanxi	PX607300	PX607325	PX628420	–	PX628400	PX628373
*Col*. *rhodoviolacea*	BJTC FM4130	China: Shanxi	PX607303	PX607324	PX628419	PX628387	PX628399	PX628372
*Col*. *sinoborealis*	BJTC FM4025	China: Shanxi	PX607307	PX607329	–	PX628390	–	PX628377
*Col*. *sinoborealis*	BJTC FM4990 (T)	China: Shanxi	PX607309	PX607331	–	–	PX628406	PX628379
*Col*. *sordida*	KUN-HKAS 106085	China: Yunnan	OP626967	OP646393	OP672227	OP871375	OP939949	OP956627
*Col*. *sordida*	SYAU-FUNGI-039	China	MK116605	MK389561	MK551219	–	–	–
*Col*. *sordida*	TENN CRG014	USA	KJ137272	–	–	KU139033	KJ137273	–
*Col*. *striaticeps*	KUN-HKAS 123129 (T)	China: Yunnan	OP626977	OP646403	OP672217	OP871385	OP939957	OP956635
***Col*. *subtropica***	**MHHNU 34153 🕱**	**China: Hunan**	**PZ159373**	**PZ176969**	–	–	–	–
***Col*. *subtropica***	**MHHNU 34154 🕱**	**China: Hunan**	**PZ159374**	**PZ176970**	–	–	–	–
***Col*. *subtropica***	**MHHNU 34191 🕱**	**China: Hunan**	**PZ159375**	**PZ176971**	–	–	–	–
***Col*. *subtropica***	**MHHNU 34194 🕱**	**China: Hunan**	**PZ159372**	**PZ176968**	–	–	–	–
*Col*. *subtropica*	KUN-HKAS 123124 (T)	China: Yunnan	OP626975	OP646401	OP672219	OP871383	OP939955	OP956633
*Col*. *subxylogena*	BJTC FM1584 (T)	China: Shanxi	PX607310	PX607332	–	–	PX628407	PX628380
*Col*. *tibetica*	KUN-HKAS 115436 (T)	China: Tibet	OP626945	OP646371	OP558066	OP763417	OP899586	OP956610
*Col*. *tomentostipes*	KUN-HKAS 123126 (T)	China: Yunnan	OP626979	OP646405	OP672215	OP871387	OP939959	OP956637
*Col*. *tuberosa*	AFTOL-ID 557	–	AY854072	AY639884	AY881025	AY857982	AY787219	–
*Col*. *urbana*	BJTC FM4970 (T)	China: Shanxi	PX607311	PX607333	PX628423	–	PX628408	PX628381
*Col*. *urbana*	BJTC FM5011	China: Shanxi	PX607312	PX607334	–	–	PX628409	PX628382
*Col*. *violea*	SYAU-FUNGI-052 (T)	China: Liaoning	MT482103	MT482097	MT510970	–	PQ817427	–
***Col*. *violea***	**MHHNU 33752**	**China: Ningxia**	**PZ159376**	**PZ176972**	**PZ173027**	**PZ173006**	–	**PZ189233**
***Col*. *violea***	**MHHNU 34607**	**China: Ningxia**	**PZ159377**	**PZ176973**	**PZ173028**	**PZ173007**	–	**PZ189234**
*Col*. *xylogena*	KUN-HKAS 54966 (T)	China: Yunnan	OP626981	OP646407	OP672233	–	OP956582	OP956640
*D*. *racemosa*	DEB5575/DED5575	–/USA	DQ825425	AF042598	KP255476	DQ825417	DQ825409	–
*L*. *cremeoinvoluta*	KUN-HKAS 122326 (T)	China: Yunnan	OR912959	OR912956	OR921064	–	–	OR921061
*L*. *densifolia*	SYAU-FUNGI-022	China	MK116588	MK278275	–	–	–	–
*L*. *mutiformis*	MHHNU 20073	China: Yunnan	OR912960	OR912957	OR921065	–	–	OR921062
*L*. *panaeola*	KUN-HKAS 105370	China	OP626982	OP646408	OP672234	OP871389	OP939961	OP956641
“*L*. *irina*”	AFTOL-ID 815	USA	DQ221109	DQ234538	DQ028591	DQ447919	DQ385885	–
*P*. *flavodepressum*	BJTC FM144 (T)	China: Shanxi	PX607315	PX607337	PX628426	–	PX628410	PX628385
*P*. *flavodepressum*	BJTC FM470	China: Shanxi	PX607316	PX607338	PX628427	PX628391	PX628411	PX628386
“*P*. *liwanense*”	HKAS 153564	China: Guangdong	NR 202931	PX857255				
*P*. *luteobrunneum*	BJTC FM3744 (T)	China: Shanxi	PX607319	PX607341	PX628430	PX628394	PX628414	–
*P*. *luteobrunneum*	BJTC FM3762	China: Shanxi	PX607320	PX607342	PX628431	PX628395	PX628415	–
*P*. *macrobasidium*	KUN-HKAS 123131 (T)	China: Gansu	NR198329	NG243793	OP672237	–	OP939963	OP956644
*P*. *metachroum*	KUN-HKAS 123133	China: Yunnan	OP626987	OP646413	OP687889	OP871393	OP939965	OP956646
*S*. *adirondackensis*	TENN 060310	USA	JX514123	JX514105	KF208440	–	JX514142	–
*S*. *alboinfundibuliformis*	KUN-HKAS 114309	China: Yunnan	OP626988	–	OP687890	OP871394	OP939966	OP956647
*S*. *albomarginata*	BJTC FM2550 (T)	China: Shanxi	PX607321	–	PX628432	PX628396	PX628416	–
*S*. *clitocyboides*	KUN-HKAS 75453	Australia	JX514118	JX514113	KF208444	–	JX514149	–
*S*. *humilis*	KUN-HKAS 74717	Sri Lanka	JX514131	JX514114	–	–	JX514150	–
*S*. *umbilicata*	KUN-HKAS 105572	China: Gansu	MZ714585	MZ714591	MZ681875	MZ681886	MZ681896	MZ681905
*S*. *umbilicata*	KUN-HKAS 77290 (T)	China: Yunnan	KF208450	KF208457	KF208438	–	KF208460	–
*Atr*. aff. *inornata*	KUN-HKAS 105559	China: Gansu	MZ714586	MZ714592	MZ681876	MZ681887	MZ681897	–
*Atr*. aff. *inornata*	KUN-HKAS 105578	China: Gansu	MZ714587	MZ714593	MZ681877	MZ681888	MZ681898	–

The newly generated sequences are shown in bold. “🕱” and “T” represent specimens from the poisoning sites and holotypes, respectively.

## Data Availability

The DNA sequences obtained in this study have been submitted to GenBank.

## Supplementary Materials

The following supporting information can be downloaded at: https://www.mdpi.com/article/10.3390/jof12080548/s1, Supplementary Material S1: Dataset I; Supplementary Material S2: Dataset II; Supplementary Material S3: Dataset III.
